# Meta-analytic evidence of elevated choline, reduced N-acetylaspartate, and normal creatine in schizophrenia and their moderation by measurement quality, echo time, and medication status

**DOI:** 10.1016/j.nicl.2023.103461

**Published:** 2023-06-27

**Authors:** Yvonne S. Yang, Jason Smucny, Huailin Zhang, Richard J. Maddock

**Affiliations:** a VISN22 Mental Illness Research, Education and Clinical Center, VA Greater Los Angeles Healthcare System, 11301 Wilshire Blvd, Los Angeles, CA 90073, USA; b Department of Psychiatry and Biobehavioral Sciences, University of California, Los Angeles, 760 Westwood Plaza, Los Angeles, CA 90095, USA; c Imaging Research Center, University of California, Davis, 4701 X Street, Sacramento, CA 95817, USA; d Department of Psychiatry and Biobehavioral Sciences, University of California, Davis, 2230 Stockton Blvd, Sacramento, CA 95817, USA; e Department of Internal Medicine, Adventist Health White Memorial, 1720 E Cesar E Chavez Ave, Los Angeles, CA 90033, USA

**Keywords:** Schizophrenia, Magnetic resonance spectroscopy, Metabolites, Measurement quality, Echo time, MRS

## Abstract

**Background:**

Brain metabolite abnormalities measured with magnetic resonance spectroscopy (MRS) provide insight into pathological processes in schizophrenia. Prior *meta*-analyses have not yet answered important questions about the influence of clinical and technical factors on neurometabolite abnormalities and brain region differences. To address these gaps, we performed an updated *meta*-analysis of N-acetylaspartate (NAA), choline, and creatine levels in patients with schizophrenia and assessed the moderating effects of medication status, echo time, measurement quality, and other factors.

**Methods:**

We searched citations from three earlier *meta*-analyses and the PubMed database after the most recent *meta*-analysis to identify studies for screening. In total, 113 publications reporting 366 regional metabolite datasets met our inclusion criteria and reported findings in medial prefrontal cortex (MPFC), dorsolateral prefrontal cortex, frontal white matter, hippocampus, thalamus, and basal ganglia from a total of 4445 patient and 3944 control observations.

**Results:**

Patients with schizophrenia had reduced NAA in five of the six brain regions, with a statistically significant sparing of the basal ganglia. Patients had elevated choline in the basal ganglia and both prefrontal cortical regions. Patient creatine levels were normal in all six regions. In some regions, the NAA and choline differences were greater in studies enrolling predominantly medicated patients compared to studies enrolling predominantly unmedicated patients. Patient NAA levels were more reduced in hippocampus and frontal white matter in studies using longer echo times than those using shorter echo times. MPFC choline and NAA abnormalities were greater in studies reporting better metabolite measurement quality.

**Conclusions:**

Choline is elevated in the basal ganglia and prefrontal cortical regions, suggesting regionally increased membrane turnover or glial activation in schizophrenia. The basal ganglia are significantly spared from the well-established widespread reduction of NAA in schizophrenia suggesting a regional difference in disease-associated factors affecting NAA. The echo time findings agree with prior reports and suggest microstructural changes cause faster NAA T2 relaxation in hippocampus and frontal white matter in schizophrenia. Separating the effects of medication status and illness chronicity on NAA and choline abnormalities will require further patient-level studies. Metabolite measurement quality was shown to be a critical factor in MRS studies of schizophrenia.

## Highlights

• Meta-analysis of MRS studies of brain NAA, creatine, and choline in schizophrenia.
• Choline increased, NAA reduced except in basal ganglia, creatine unchanged.
• NAA was more reduced with longer echo times in some regions.
• Larger effect sizes for NAA and choline in more medicated samples in some regions.
• Choline more elevated and NAA more reduced with better measurement quality.

## 1 Introduction

Schizophrenia is within the top fifteen causes of disability worldwide and represents an excess economic burden of $330.6B in the United States in 2019 (Kadakia et al., 2022, Vos et al., 2017). Over a century after its first description in 1911 by Eugen Bleuler, its physiological underpinnings are largely unknown. Magnetic resonance spectroscopy (MRS) can provide insights into the biological mechanisms of schizophrenia by providing information about metabolites important to brain function. Compared to other approaches, MRS has several useful advantages: it can be performed in vivo, is non-invasive, can be used translationally in both preclinical and clinical studies, and is readily available on most standard MRI scanners.

The three metabolite signals measured most consistently with MRS are N-acetylaspartate (NAA), choline-containing compounds, and total creatine (creatine plus phosphocreatine). Despite the relative ease of measuring these singlet peaks, which do not require special methods such as spectral editing, there continues to be lack of consensus regarding changes in these three metabolites in schizophrenia. These inconsistencies have been attributed to variations in equipment (magnetic field strength, manufacturer), acquisition parameters, analysis methodology, variable data quality, brain region, clinical population (early vs chronic), and small sample sizes. This *meta*-analysis attempts to address some of these outstanding questions.

### 1.1 NAA

The most consistent MRS finding in schizophrenia has been decreased NAA in patients compared to healthy control subjects, including in several large *meta*-analyses and systematic reviews (Bracken et al., 2013, Iwata et al., 2018, Kraguljac et al., 2012, Steen et al., 2005, Whitehurst et al., 2020). NAA is thought to be a marker of neuronal integrity (Maddock and Buonocore, 2012) a marker of mitochondrial energy production (Gonçalves et al., 2015), and a factor in oligodendrocyte myelin and acetate metabolism (Chakraborty et al., 2001). Decreased NAA has been associated with both neurodegeneration and neuroinflammation in studies of other disorders, including findings of reduced levels in Alzheimer’s disease, Huntington disease, and multiple sclerosis (Paslakis et al., 2014, Tsai and Coyle, 1995). A previous *meta*-analysis of schizophrenia studies suggests that the reduction in NAA may be progressive over the course of the illness and not observed in unmedicated patients (Whitehurst et al., 2020). Decreased NAA has been noted to correlate with severity of symptoms in chronic patients (Premkumar et al., 2010) and those at high risk for schizophrenia (Liemburg et al., 2016). Therefore, changes in NAA may be related to the pathophysiology of schizophrenia, and NAA levels have potential to be a biomarker of therapeutic targets in schizophrenia. The finding of lower NAA in schizophrenia, however, has been called into question by reports suggesting that faster T2 relaxation of NAA in schizophrenia may complicate attempts to estimate actual NAA content in this disorder, particularly in frontal white matter and the hippocampal region (Bracken et al., 2013, Kuan et al., 2021, Tunc-Skarka et al., 2009). To help address some of the open questions about NAA, this updated *meta*-analysis examined the magnitude and reliability of reduced NAA in schizophrenia as well as potential moderator effects of medication status, phase of illness and echo time on this abnormality.

### 1.2 Choline

Choline-containing compounds measured by MRS (mainly phosphocholine and glycerophosphocholine) are important in phospholipid metabolism (Klein, 2000) and for providing methyl groups for DNA methylation (Saito et al., 2022). Increased choline measured with MRS is thought to reflect increased neuronal membrane turnover or breakdown and reflect the density of cell membranes in a voxel (Maddock and Buonocore, 2012). Choline has also been associated with glial activation due to neuroinflammation (Chelala et al., 2020, Dahmani et al., 2021, Urenjak et al., 1993). Prior MRS studies of choline in schizophrenia have been inconsistent, with some evidence for elevated choline (Romeo et al., 2020) and other evidence that choline is unchanged (Kraguljac et al., 2012). The current report will provide an updated *meta*-analysis of this literature with particular attention to moderators that may influence the finding of choline abnormalities in schizophrenia.

### 1.3 Creatine

The creatine signal measured by proton MRS on clinical scanners represents the sum of phosphocreatine and creatine. These two forms of creatine are an essential part of energy metabolism infrastructure in all cells, including all neuronal and glial cells in the brain (Maddock and Buonocore, 2012, Szulc et al., 2011). While the proportion of creatine and phosphocreatine varies as a function of the energy status of a cell, the total amount of creatine is thought to be relatively stable across time and across different brain areas (Maddock and Buonocore, 2012). As such, creatine values are often used for normalization of other metabolite levels. This practice, however, has been called into question for the study of brain metabolites in schizophrenia due to reports of decreased creatine in the illness compared to healthy controls (Ongür et al., 2009). Only two prior studies have reported *meta*-analytic comparisons of creatine levels in patients and controls (Kraguljac et al., 2012, Steen et al., 2005). Although both reported finding no significant differences, the most recent *meta*-analysis was conducted>10 years ago, and many investigators in the field continue to voice a concern that creatine levels may be abnormal in schizophrenia. The current report aims to provide an updated analysis of brain creatine levels in schizophrenia and offer some guidance as to the suitability of creatine-normalization in studies of this disorder.

### 1.4 Moderating factors

A recent *meta*-analysis showed that metabolite measurement quality significantly moderated effect sizes in MRS studies of glutamate in schizophrenia. Smucny et al. (2021) found that the pooled data from studies with better measurement quality showed a significant reduction in medial prefrontal cortex glutamate with minimal heterogeneity, while pooled data from studies with lower measurement quality showed no significant difference in glutamate and substantial heterogeneity. Glutamate is a j-coupled multiplet that can be challenging to measure on clinical scanners. NAA, choline and creatine are singlets that are considered reliably measurable. In this *meta*-analysis we will examine whether measurement quality similarly moderates the effect sizes for schizophrenia patient versus control differences in NAA, creatine, and choline. Measurement quality metrics include coefficient of variation (COV) of metabolite values, Cramer-Rao Lower Bound (CRLB) of metabolite model fits, metabolite singlet line width (FWHM), and signal to noise ratio (SNR) of acquired spectra. In addition to examining group differences across several different brain areas between patients and healthy volunteers and the influence of measurement quality metrics, we also examined the moderating effects of clinical characteristics (patient age, patient sex, medication status, and phase of illness) and technical parameters (field strength, echo time (TE) and normalization method).

## 2 Methods

### 2.1 Literature review

**Fig. 1:**
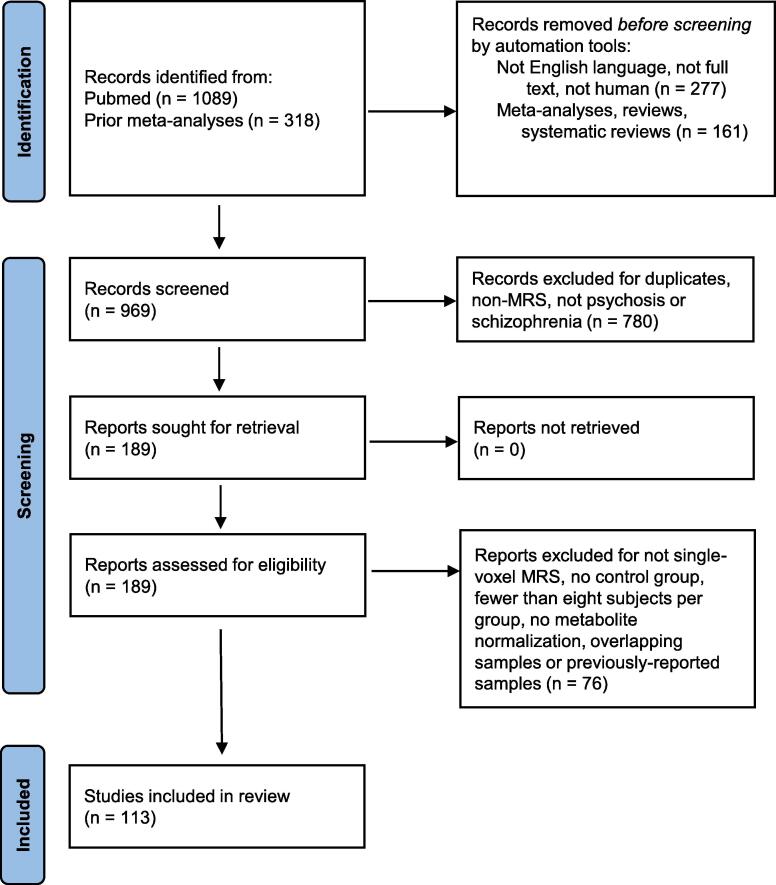
PRISMA Consort Flow Diagram.

The literature review for this *meta*-analysis was performed according to PRISMA guidelines (Page et al., 2021). Studies from three previous MRS *meta*-analyses (Bracken et al., 2013, Iwata et al., 2018, Whitehurst et al., 2020) were included and a PubMed database search was performed to identify more recent studies. A search with the term ((schizophreni* OR psychos* OR Schizoaffective) AND (magnetic resonance spectroscopy OR MRS OR Spectroscopy)) from January 1, 2019 to March 31, 2021 yielded an initial 1089 studies. After applying the criteria of full-text, English-language, and human studies, 812 studies remained. After excluding reviews, systematic reviews, and *meta*-analyses, 651 studies remained. These studies were combined with the 318 studies from the three previously-published *meta*-analyses and screened for non-MRS studies, duplicates, and non primary psychotic disorder diagnoses, leaving 189 studies sought for retrieval (Fig. 1).

### 2.2 Data extraction

For the brain regions examined, authors M.Z. extracted and Y.Y., J.S., and R.M. verified data. Extracted data included final sample sizes for each metabolite, brain region studied, means & standard deviations (SDs) of NAA, choline and creatine, means & SDs of metabolite CRLBs, means & SDs of line width (quantified as full-width at half maximum (FWHM) of singlet peaks) and means & SDs of signal to noise ratio (SNR). We also extracted field strength, echo time (TE), metabolite normalization method (water or creatine), percent of patients of each sex, mean duration of illness, mean patient age, and medication status information.

### 2.3 Eligibility criteria

Because the MRS literature on schizophrenia is now quite extensive, we adopted relatively conservative eligibility criteria to reduce heterogeneity and ensure data from the same subjects were not included more than once. All studies were scrutinized for samples that overlapped with other studies. When overlapping samples were identified, only data from the single study with the largest sample were included. When studies reported separately on multiple patient and control groups, they were treated as independent datasets. When multiple patient groups were compared to a single control group, the patient groups were combined and treated as a single dataset. For longitudinal studies, only the values given for the first time point were included. When metabolite values normalized to both water and creatine were reported, the normalization method producing the lowest coefficient of variation for the metabolite (COV) averaged across groups was used. Studies were excluded if normalization method was not reported, not performed, or did not use either water or creatine. When bilateral metabolite values were reported, only the hemisphere most commonly studied for that region was included (left for all regions). When studies reported on two nearby voxels within the same region, the voxel with higher metabolite COV was excluded. When studies separately reported metabolite values acquired at different TEs, only values from the shortest TE were used. Only studies using single-voxel localization methods generating 1-D spectra using a single TE were included.

### 2.4 Data analysis

As previously described (Smucny et al., 2021), effect size for each dataset was calculated as Hedge’s *g*, which corrects for small sample sizes (Hedges and Olkin, 2014). An inverse variance-weighted, random effects model was utilized to calculate the *meta*-analysis pooled effect size. To determine significance, tau^2^ was calculated by the restricted maximum likelihood method. Meta-analyses and moderator analyses were conducted with the R-based software JASP (JASP Team, 2022). Heterogeneity across studies was quantified as I^2^, and a chi-square test of the Q statistic tested for significant non-homogeneity. Since this report focuses on how moderators such as measurement quality, acquisition parameters and clinical parameters influence pooled effect sizes, *meta*-analyses were performed only in brain regions for which ≥ 10 datasets were available (for at least one metabolite), as recommended by the Cochrane Handbook (Higgins et al., 2019).

For metabolites found to be significantly different in schizophrenia in any brain region, we calculated the patient versus control weighted mean percent difference across datasets for all regions. This was calculated as follows:

$$\frac{\sum_{i=1}^{k}\left[\left(pt\overset{¯}{x_{i}}-con\overset{¯}{x_{i}}\right)/\left(con\overset{¯}{x_{i}}\right)\right]*\left(pt\;N_{i}+con\;N_{i}\right)}{\sum_{i=1}^{k}\mathrm{pt}\;N_{i}+con\;N_{i}}$$

where k = the number of datasets in a *meta*-analytic comparison, $pt\;\overset{¯}{x_{i}}$ = mean patient metabolite value in each dataset, $con\;\overset{¯}{x_{i}}$ = mean control metabolite value in each dataset, $pt\;N_{i}$ = number of patients in each dataset, and $con\;N_{i}$ = number of controls in each dataset.

#### 2.4.1 Moderation of effects sizes by metabolite measurement quality

As in our previous *meta*-analysis of glutamate measurements in schizophrenia (Smucny et al., 2021), we hypothesized any true group difference in metabolite values would be most evident in studies with relatively better-quality metabolite measurements. For the coefficient of variation (COV) of metabolite values, we calculated the average COV (as SD/mean) of the patient and control groups. For FWHM and SNR, we calculated the mean + 2 SD (mean – 2 SD for SNR) for the patient and control groups and then averaged those values. Approximately 97.5% of subjects would have quality values below this level (above for SNR) for each study. If only the mean was reported, SD would be imputed using the median of the SD/mean ratios from all other studies reporting both mean and SD. For CRLB, very few studies reported the SD, so only the mean CRLB across patient and control groups was calculated. Consistent with our prior *meta*-analysis (Smucny et al., 2021), we reasoned that the relationship between measurement quality and effect size would be logistic (sigmoid), rather than linear. That is, we expected there would be an empirically identifiable quality threshold beyond which pooled effect sizes would become larger and more consistent. Formally, we hypothesized there was a quality threshold T, for which the *meta*-analytic result would be significantly stronger in studies surpassing T than for those falling short of T. To identify the quality threshold, we plotted the inverse variance-weighted pooled effect sizes (as *g*) from moving sub-*meta*-analyses running from the lowest to the highest quality studies for each quality metric (analogous to a moving average), as previously described (Smucny et al., 2021). The number of studies included in each moving sub-*meta*-analysis (k’) was the greater of 7 or k/5 (where k = the total number of studies reporting the quality metric). A best-fitting, 4-parameter, logistic function was fit to these pooled effect sizes (Phillips, 2016), and the resulting equation was used to identify the inflection point, which defined the quality threshold T (Smucny et al., 2021). When the logistic fit was significant and the inflection point was within the range of the included studies, the inflection point was used to stratify the studies into low- and high-quality subgroups for each quality metric (see Supplemental Methods). Directly comparing these subgroups tested our hypothesis that the pooled effect size would be stronger in studies with higher quality measurements. When the quality subgroups were significantly different, secondary *meta*-analyses were conducted on the individual subgroups. Since this procedure requires a minimum of approximately 14 studies (twice the minimum number in a moving sub-*meta*-analysis of 7 studies) to reliably identify higher and lower quality subgroups, it was only performed when the number of datasets reporting the quality metric was ≥ 14. In our previous *meta*-analysis of glutamate in schizophrenia, COV was available as a measurement quality metric for all studies, while other quality metrics, including CRLB, FWHM and SNR, were omitted from the reports of many studies. Thus, we elected to use COV as the measurement quality metric for hypothesis testing in the primary analyses for each regional metabolite. When ≥ 14 datasets reported other quality metrics, the moderating effects of measurement quality as reflected by these metrics were examined in secondary analyses.

#### 2.4.2 Moderation of effect sizes by technical and clinical factors

Field strength (<3T versus ≥ 3 T), echo time (as log TE) and normalization method (water or creatine) were examined as potential moderators for each regional metabolite using subgroup analyses or *meta*-regression, as appropriate. Clinical variables, including mean patient age, percent males in patient group, and percent of patients on antipsychotic medication (as *meta*-regressors) and recent onset versus chronic psychosis (as a subgroup analysis comparing datasets with mean duration of illness < 36 months versus ≥ 36 months) were also examined.

#### 2.4.3 Small study bias, robustness, and outlier datasets

For all *meta*-analyses, small study bias was tested using the Egger regression test for funnel plot asymmetry (JASP Team, 2022). If the Egger test was significant, the small study responsible for the biasing effect was identified graphically and removed. This occurred in two instances, and in both cases only one study was removed and the pattern of results either remained the same or a trend became a significant effect. For all significant *meta*-analytic results, leave-one-out sensitivity analysis was performed to examine the robustness of the *meta*-analytic result. When leaving out any one study rendered the *meta*-analytic result non-significant, it was considered not robust. If any individual dataset’s 95% confidence interval (CI) did not overlap with the overall 95% CI for any *meta*-analysis, such studies were considered outliers and the results of the *meta*-analysis were also examined with these outlier studies excluded. If exclusion of outliers changed the statistical significance of an analysis or changed a significant effect size by ≥ 1/3 (33%), then the results with outliers removed were also reported. Note that outlier status was always relative to the CIs of a specific *meta*-analysis or moderator analysis.

## 3 Results

After applying the eligibility criteria, 113 publications reporting on 366 non-overlapping regional metabolite datasets were included in the final sample (Aoyama et al., 2011, Auer et al., 2001, Bartha et al., 1997, Bartha et al., 1999, Bartolomeo et al., 2019, Başoğlu et al., 2006, Batalla et al., 2015, Bertolino et al., 2000, Brandt et al., 2016, Buckley et al., 1994, Bustillo et al., 2001, Bustillo et al., 2002, Bustillo et al., 2008, Bustillo et al., 2010 Bustillo et al., 2014, Cecil et al., 1999, Chang et al., 2007, Chiappelli et al., 2015, Chiu et al., 2018, Choe et al., 1994, Coughlin et al., 2015, de la Fuente-Sandoval et al., 2018, Delamillieure et al., 2000a, Delamillieure et al., 2002, Demjaha et al., 2014, Egerton et al., 2018, Fannon et al., 2003, Fujimoto et al., 1994, Fukuzako, 2000, Fukuzako et al., 1995, Galińska et al., 2009, Gallinat et al., 2016, Goldstein et al., 2015, Granata et al., 2013, Hagino et al., 2002, Hasan et al., 2014, He et al., 2012, Heimberg et al., 1998, Huang et al., 2017, Hutcheson et al., 2012, Iwata et al., 2019, Jessen et al., 2006, Jessen et al., 2013, Kegeles et al., 2012, Kirtaş et al., 2016, Klär et al., 2010, Korenic et al., 2020, Kraguljac et al., 2019, Kumar et al., 2020, Larabi et al., 2017, Legind et al., 2019, Liemburg et al., 2016, Maier et al., 2000, Malchow et al., 2013, Marsman et al., 2014, Merritt et al., 2019, Miyaoka et al., 2005, Molina et al., 2005, Molina et al., 2007, Natsubori et al., 2014, van Elst et al., 2005, Delamillieure et al., 2000b, Lutkenhoff et al., 2010 Nenadic et al., 2015, Ohara et al., 2000, Ohrmann et al., 2008, Olbrich et al., 2008, Omori et al., 2000, Öngür et al., 2010, Ota et al., 2012, Ota et al., 2015, Pillinger et al., 2019, Plitman et al., 2016, Plitman et al., 2018, Posporelis et al., 2018, Premkumar et al., 2010, Reid et al., 2010, Reid et al., 2013, Reid et al., 2016, Reid et al., 2018, Rowland et al., 2009, Rowland et al., 2013, Rowland et al., 2015, Rowland et al., 2016, Rüsch et al., 2008, Shakory et al., 2018, Shioiri et al., 1996, Shirayama et al., 2010, Sigmundsson et al., 2003, Singh et al., 2018, Sivaraman et al., 2018, Stanley et al., 2007, Steel et al., 2001, Szulc et al., 2006, Szulc et al., 2011, Tarumi et al., 2020, Taylor et al., 2017, Tayoshi et al., 2009, Terpstra et al., 2005, Théberge et al., 2007, Tibbo et al., 2013, Tunc-Skarka et al., 2009, Uhl et al., 2011, Venkatraman et al., 2006, Wang et al., 2019, Wijtenburg et al., 2017, Wood et al., 2007, Yamasue et al., 2002, Yamasue et al., 2003, Yasukawa et al., 2005 Yurgelun-Todd et al., 1996, Zabala et al., 2007, Zong et al., 2015). A dataset was defined as a patient-control comparison for one brain area and one metabolite. Some studies contributed datasets for all three metabolites in multiple regions, while others did so for only one or two metabolites in a single region. Four publications reported multiple non-overlapping patient-control comparisons, such as older and younger patients with matched older and younger control samples. Of the 366 total datasets, 154 reported brain NAA, 131 reported brain choline and 81 reported brain creatine. These included metabolite data from the medial prefrontal cortex (MPFC), dorsolateral prefrontal cortex (DLPFC), hippocampus (HC), thalamus, basal ganglia (BG), and frontal white matter (FrWM). Other brain regions were represented by<10 datasets and were not included in the *meta*-analyses.

### 3.1 Primary meta-analytic results by metabolite

#### 3.1.1 NAA is decreased across numerous brain areas in patients compared to controls

**Fig. 2:**
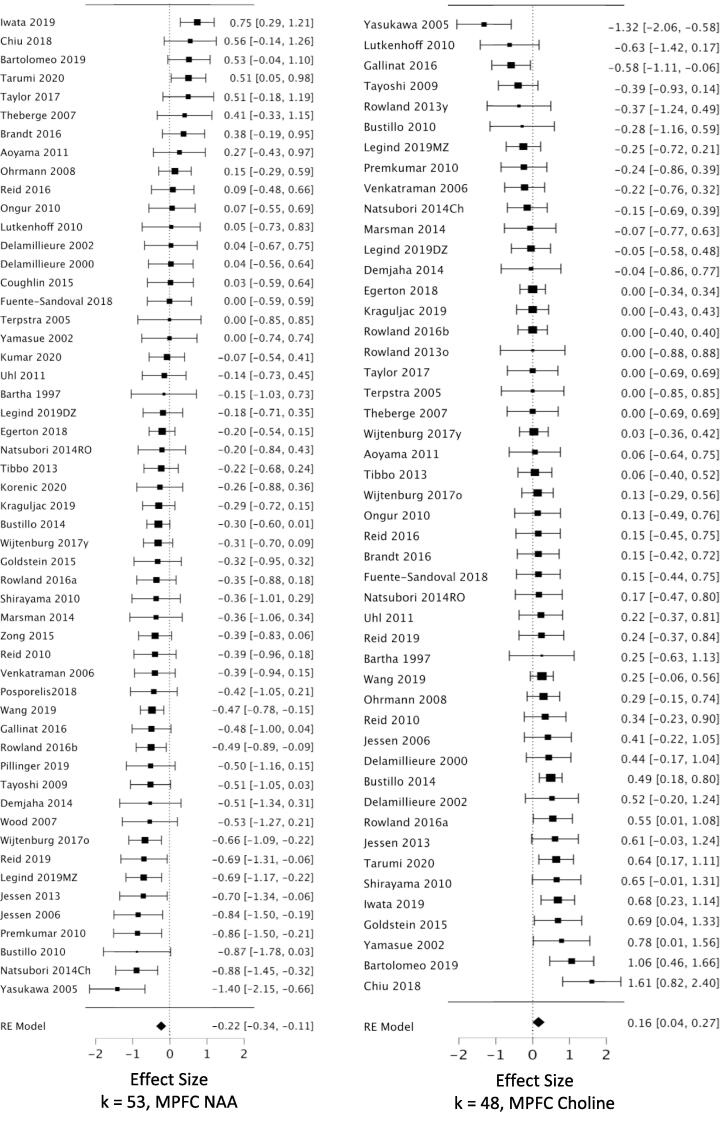
Forest plots of effect sizes for medial prefrontal cortex NAA and choline datasets.

**Fig. 3:**
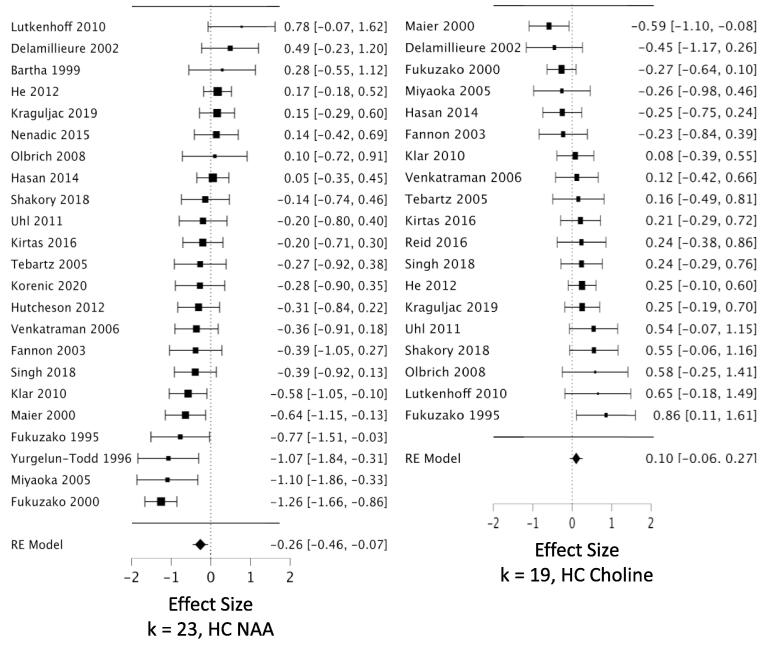
Forest plots of effect sizes for hippocampal NAA and choline datasets.

**Fig. 4:**
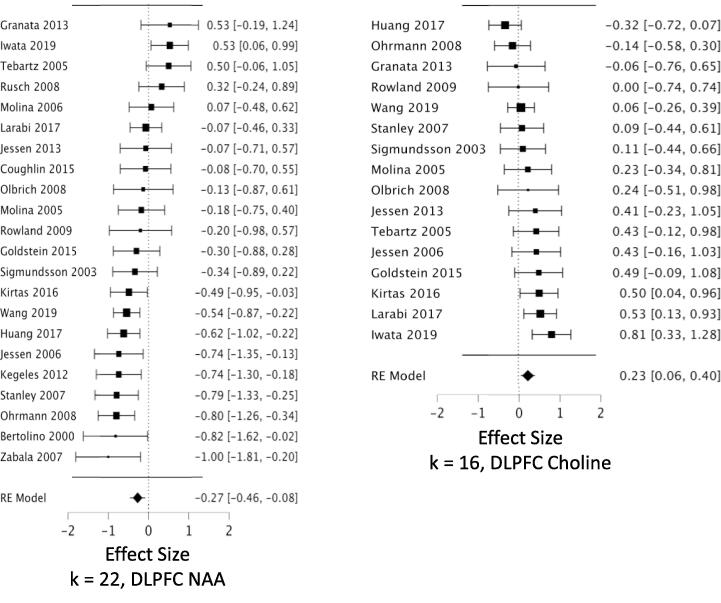
Forest plots of effect sizes for dorsolateral prefrontal cortex NAA and choline datasets.

**Fig. 5:**
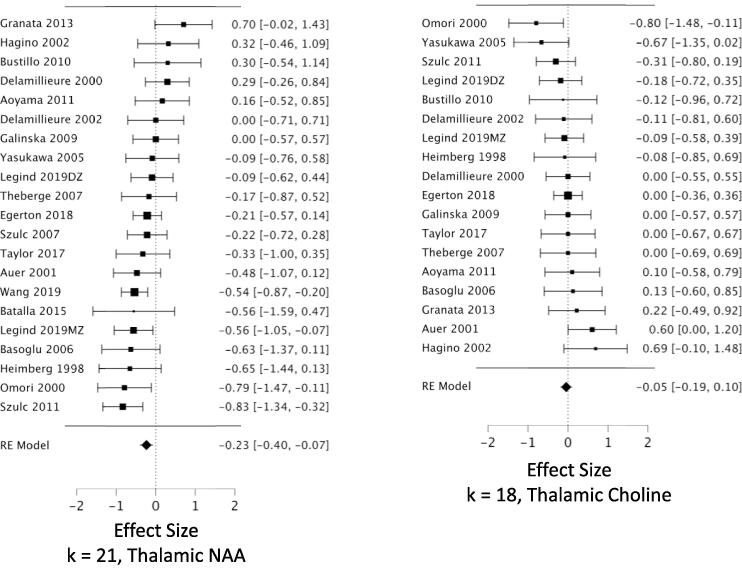
Forest plots of effect sizes for thalamic NAA and choline datasets.

**Fig. 6:**
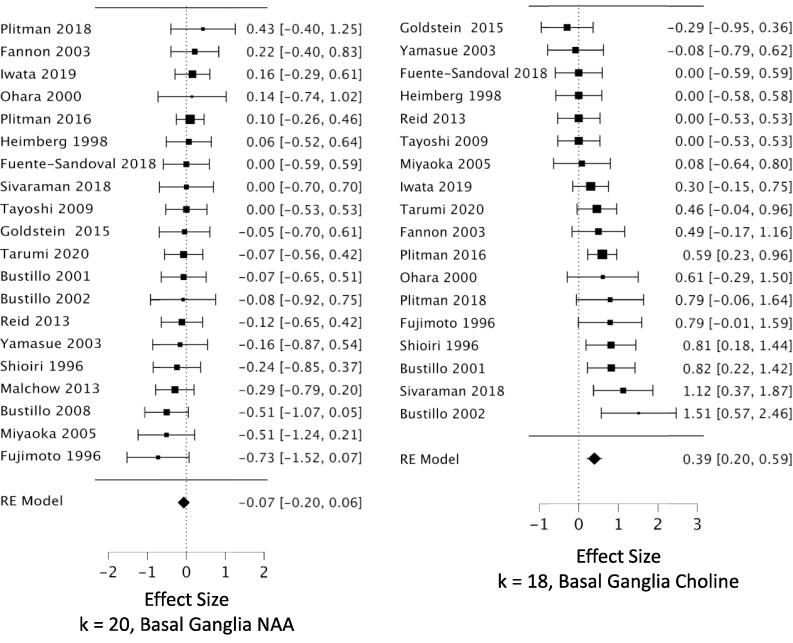
Forest plots of effect sizes for basal ganglia NAA and choline datasets.

**Fig. 7:**
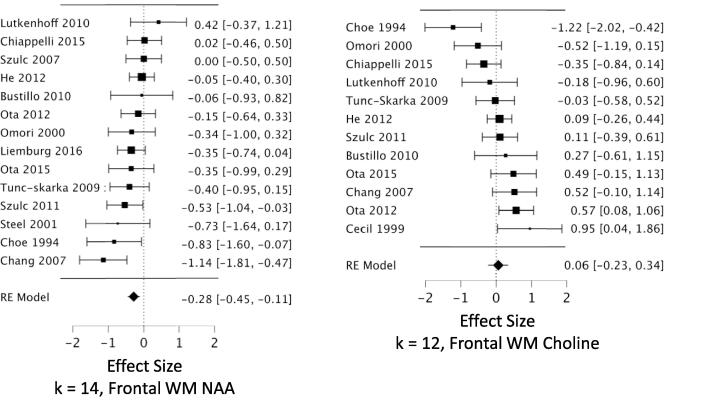
Forest plots of effect sizes for frontal white matter NAA and choline datasets.

*MPFC:* Across 53 datasets reporting MPFC NAA, we found a small but highly reliable reduction in NAA in schizophrenia (*g* = -0.22; CI, −0.11 to −0.34; Q = 15.7, df = 1, p =.00007; heterogeneity: I^2^ = 52.2%) (Fig. 2, Table 1). The results were free of small study bias and robust to leave-one-out analysis. Seven datasets in this analysis were identified as outliers (see Methods 2.4.3). When these were removed, the effect size for NAA became stronger (*g* = -0.31) (Table 1). *Hippocampus:* Our analysis revealed a significant reduction in hippocampal NAA across 23 datasets (*g* = -0.26; CI, −0.07 to −0.46; Q = 6.9, df = 1, p =.0088; heterogeneity: I^2^ = 63.8%) (Fig. 3, Table 1). The result was free of small study bias, robust to leave-one-out analysis, and was unchanged when one outlier was removed. *DLPFC:* Our analysis found a significant reduction in NAA across 22 datasets (*g* = -0.27; CI, −0.08 to −0.46; Q = 7.6, df = 1, p =.0059; heterogeneity: I^2^ = 63.2%) (Fig. 4, Table 1). When the two outlier datasets were removed, the effect size for reduced NAA became more negative (g = -0.36). The results were free of small study bias and robust to leave-one-out analysis. *Thalamus:* Our analysis showed a small but significant NAA reduction in thalamus across 21 datasets (*g* = -0.23; CI, −0.07 to −0.40; Q = 7.7, df = 1, p =.0055; heterogeneity: I^2^ = 37.3%) (Fig. 5, Table 1). The significant effect was free of small study bias, robust to leave-one-out analysis, and unchanged when one outlier was removed (Table 1). *Basal ganglia:* Across 20 datasets reporting on the basal ganglia, we observed no significant difference in NAA between the patient and control groups (*g* = -0.07; CI, +0.06 to −0.20; Q = 01.0, df = 1, p =.313; heterogeneity: I^2^ = 0.0%) (Fig. 6, Table 1). *FrWM:* In the primary *meta*-analytic model for NAA, one study was excluded for small study bias. Across the remaining 14 datasets, NAA was significantly reduced NAA in patients compared to control subjects (*g* = -0.30; CI, -0.12 to -0.48; Q = 10.7, df = 1, p =.001; heterogeneity: I^2^ = 30.1%) (Fig. 7, Table 1). This significant reduction was robust to leave-one-out analysis, and there were no outlier studies.

**Table 1:** Primary Meta-analytic Results for Three Metabolite Singlets in Six Brain Regions.

	Region	K	Pts	HC	Effect Size (95% CI)	P value	Percent Diff a Pt - HC	Heterogeneity I^2^, % P value		number outliers (effect?) b
NAA	MPFC	53	1527	1435	**-0.22 (-0.34 to -0.11)**	**0.00007**	**−2.2%**	52.2	<0.001	7 (Y)
	-Excl. outliers	46	1295	1286	**-0.32 (-0.39 to -0.24)**	**<0.00001**	**−3.2%**	0.0	0.47	
	HC	23	615	611	**-0.26 (-0.46 to -0.07)**	**0.0088**	**−4.4%**	63.8	<0.001	1 (N)
	DLPFC	22	728	633	**-0.27 (-0.46 to -0.08)**	**0.0059**	**−4.2%**	63.2	<0.001	2 (Y)
	-Excl. outliers	20	643	574	**-0.36 (-0.53 to -0.19)**	**0.00002**	**−5.7%**	45.0	0.015	
	Thal	21	557	506	**-0.23 (-0.40 to -0.07)**	**0.0055**	**−2.0%**	37.3	0.042	1 (N)
	BG	20	563	414	-0.07 (-0.20 to + 0.06)	0.31	−1.3%	0.0	0.88	0
	FrWM	14^c^	455	345	**-0.28 (-0.45 to -0.11)**	**0.0012**	**−3.4%**	21.8	0.14	1 (N)
Choline	MPFC	48	1380	1279	**+0.16 (+0.04 to + 0.27)**	**0.0084**	**+3.0%**	51.0	<0.001	4 (N)
	HC	19	553	514	+0.10 (-0.06 to + 0.27)	0.21	+2.9%	38.7	0.037	1 (N)
	DLPFC	16	593	504	**+0.23 (+0.06 to + 0.40)**	**0.0067**	**+3.0%**	41.1	0.064	0
	Thal	18	423	402	-0.05 (-0.19 to + 0.10)	0.51	−0.3%	0.0	0.40	0
	BG	18	489	367	**+0.39 (+0.20 to + 0.59)**	**0.00006**	**+6.9%**	42.4	0.023	0
	FrWM	12	307	293	+0.06 (-0.23 to + 0.34)	0.69	+1.2%	63.5	0.004	1 (N)
Creat	MPFC	38	1104	1056	-0.04 (-0.17 to + 0.09)	0.53		51.7	<0.001	3 (N)
	HC	7	138	189	0.00 (-0.33 to + 0.33)	0.99		51.8	0.051	0
	DLPFC	11	422	389	-0.01 (-0.19 to + 0.16)	0.88		27.4	0.25	0
	Thal	9	191	200	+0.02 (-0.18 to + 0.23)	0.83		0.0	0.96	0
	BG	11	343	250	+0.13 (-0.03 to + 0.30)	0.12		0	0.77	0
	FrWM	5	104	107	+0.14 (-0.23 to + 0.51)	0.45		39.7	0.15	0

Abbreviations: MPFC, medial prefrontal cortex; HC, hippocampus; DLPFC, dorsolateral prefrontal cortex; Thal, thalamus; BG, basal ganglia; FrWM, frontal white matter; K = number of datasets; Pts = patients; HC = healthy controls; Excl. = after excluding.

a Weighted mean percent difference between patients and controls. See section 2.4 in text.

b Number of outlier datasets for which the 95% CI does not overlap the 95% CI of the pooled data. (Y) or (N) = exclusion of outliers DOES (Y) or DOES NOT (N) change the statistical significance or change a significant effect size by ≥ 1/3.

c One study identified as a source of small study bias was excluded from this model.

#### 3.1.2 Choline is increased in several brain areas in patients compared to controls

We observed increased choline in patients compared to controls in MPFC, DLPFC, and basal ganglia. *MPFC:* Across 48 datasets reporting on MPFC choline, we found a small but significant elevation in choline in schizophrenia (*g* = +0.16; CI, +0.27 to + 0.04; Q = 7.0, df = 1, p =.0084; heterogeneity: I^2^ = 51.0%) (Fig. 2, Table 1). The effects were free of small study bias, robust to leave-one-out analysis, and robust to the exclusion of outliers (Table 1). *Hippocampus:* Across 19 datasets, the difference in hippocampal choline between patients and controls was not significant (*g* = +0.104; CI, +0.27 to −0.06; Q = 1.6, df = 1, p =.21; heterogeneity: I^2^ = 38.7%) (Fig. 3, Table 1). *DLPFC:* We found a small but significant elevation in DLPFC choline in schizophrenia compared to controls across 16 datasets (*g* = +0.23; CI, +0.40 to + 0.06; Q = 7.3, df = 1, p =.0067; heterogeneity: I^2^ = 41.1%) (Fig. 4, Table 1). The significant elevation was free of small study bias and robust to leave-one-out analysis. *Thalamus:* Across 18 datasets, we found no significant difference in thalamic choline between patients and control participants (*g* = -0.05; CI, +0.10 to −0.19; Q = 0.4, df = 1, p =.53; heterogeneity: I^2^ = 0.0%) (Fig. 5, Table 1). *Basal ganglia:* We found a significantly elevated choline in basal ganglia of patients compared to controls across 18 datasets (*g* = +0.395; CI, +0.59 to + 0.20; Q = 16.2, df = 1, p =.00006; heterogeneity: I^2^ = 42.4%) (Fig. 6, Table 1). This finding was robust to leave-one-out analysis and contained no outlier datasets. *FrWM:* Across 12 datasets, we found no significant difference in choline between patients and controls (*g* = +0.06; CI, +0.34 to -0.27; Q = 0.2, df = 1, p =.69; heterogeneity: I^2^ = 63.5%) (Fig. 7, Table 1). Removal of the single outlier increased the effect size but the difference remained non-significant (*g* = +0.14; CI, +0.37 to -0.09; Q = 1.4, df = 1, p =.23).

#### 3.1.3 Creatine was not different between patients and controls across all brain areas

Across 81 datasets reporting creatine in at least one brain area, we did not identify any patient-control differences in any brain area. *MPFC:* Across 38 datasets reporting MPFC creatine, we observed no significant difference between schizophrenia patients and healthy volunteer subjects (Table 1, Supplemental Figures). *Hippocampus:* An exploratory analysis of the 7 datasets reporting hippocampal creatine found no significant difference between schizophrenia patients and healthy volunteer participants (Table 1, Supplemental Figures). *DLPFC:* No significant difference in creatine was observed across 11 datasets comparing schizophrenia patients and healthy volunteers (Table 1, Supplemental Figures). *Thalamus:* An exploratory analysis of 9 datasets found no significant difference in creatine between schizophrenia patients and control participants (Table 1, Supplemental Figures). *Basal ganglia:* Across 11 datasets, we found no significant difference in creatine between people with schizophrenia and healthy volunteers (Table 1, Supplemental Figures). *FrWM:* An exploratory analysis of 5 datasets reporting creatine found no significant difference between patients and healthy volunteers (Table 1, Supplemental Figures).

### 3.2 Moderating effects of metabolite measurement quality

Primary testing of hypotheses about measurement quality were performed using COV, as this metric was available for all studies. As described in Methods, when the best-fitting logistic transform of a moving sub-*meta*-analysis was significant and identified an inflection point within the range of our datasets, the inflection point was used to dichotomize the datasets into empirically-defined lower COV and higher COV subgroups for comparison. When sufficient data were available, similar analyses were performed using CRLB, FWHM, and SNR.

#### 3.2.1 Measurement quality moderated choline and NAA effect sizes in MPFC

**Fig. 8:**
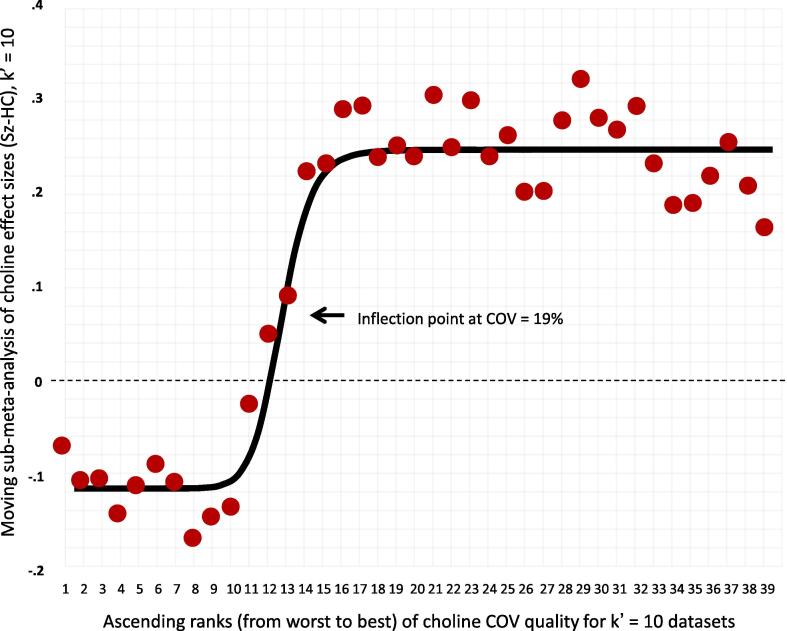
Stronger evidence for elevated MPFC choline in schizophrenia in datasets with lower coefficients of variation (COVs). Red circles represent the moving sample pooled effect size from 10 datasets in ascending ranks of COV from poorer (left) to better COV (right) along the x-axis. Circle #39 is the effect size for ten datasets ranked 30–39 (lowest COVs). Circle #1 is for datasets ranked 1–10 (total k = 48). Black line is best-fitting logistic function. Inflection point is threshold separating low- and high-quality datasets at COV = 19%. (For interpretation of the references to colour in this figure legend, the reader is referred to the web version of this article.)

Significant and robust moderation of pooled effect sizes by measurement quality was only observed for studies of the MPFC. This effect was most evident for choline-containing compounds in the MPFC. A highly significant fit was observed for the logistic transform of the COV-ranked sub-*meta*-analyses (r^2^ = 0.95, p <.00001, inflection point at COV = 19%, Table 2), with larger choline effect sizes in studies with COV ≤ 19% (Fig. 8). While choline was elevated across all datasets in this *meta*-analysis, the effect was moderated by COV quality subgroups defined by this inflection point. Across all 48 datasets, the subgroup comparison was significant (p =.034), but not robust to leave-one-out sensitivity analysis. After exclusion of four outlier datasets, however, this significant effect was robust (p =.011, Table 2). Choline was significantly elevated across the 36 datasets with COV ≤ 19% (*g* = +0.22, p =.00015), while differences between people with schizophrenia and controls were non-significant across 12 datasets with COV > 19% (*g* = -0.09, p =.58) (Table 2).

**Table 2:** Significant Quality Metric Moderators.

	Subgroups	K	Pts	HC	Effect Size with (95% CI), or r^2^ and IP	P value	Percent Diff a Pt - HC	Heterogeneity I^2^, % P value	number outliers (effect?) b
MPFC Choline	COV logistic fit	48	1380	1279	**r^2^ = 0.95, IP = 19%**	**<0.00001**	n/a		
	Lo vs Hi COV	48			Higher Chol with < COV	0.034	n/a	48.6 < 0.001	4 (Y)^c^
	-Excl Outliers	44	1283	1197	Higher Chol with < COV	**0.011**	n/a	13.0 0.41	
	Low cov grp	36	1121	1041	**+0.22 (+0.11 to + 0.33)**	**0.00015**	**+3.6%**	36.1 0.012	2 (N)
	Hi cov grp	12	259	238	-0.092 (-0.42 to + 0.23)	0.58	+0.8%	66.9 < 0.001	2 (N)
	*All studies*	*48*			***+0.16 (-0.04 to + 0.27)***	***0.0084***	***+3.0%***	*51.0 < 0.001*	*4 (N)*
									
	CRLB logistic fit	21	719	736	**r^2^ = 0.59, IP = 3.0**	**0.016**	n/a		
	Lo v Hi CRLB	21			Higher Chol with < CRLB	**0.018**	n/a	0.0 0.11	1 (N)
	Lo CRLB grp	7	303	277	+0.29 (+0.11 to + 0.46)	**0.0012**	**+4.0%**	5.9 0.45	0
	Hi CRLB grp	14	416	459	+0.031 (-0.10 to + 0.17)	0.66	+0.7%	0.0 0.07	1 (N)
MPFC NAA	SNR logistic fit	27	969	945	**r^2^ = 0.76, IP = 12.5**	**<0.00001**	n/a		
	Hi vs Lo SNR	27			Lower NAA with > SNR	**0.0064**	n/a	40.3 0.023	2 (N)
	Hi SNR grp	15	597	649	**-0.37 (-0.48 to -0.26)**	**<0.00001**	**−2.9%**	0.0 0.76	0
	Lo SNR grp	12	372	296	-0.025 (-0.29 to + 0.24)	0.86	−0.4%	63.4 0.001	0
	*All studies*	*53*	1527	1435	***-0.225 (-0.34 to -0.11)***	***0.00007***	***−2.2%***	*52.2 < 0.001*	*7 (Y)*

Abbreviations: COV, coefficient of variation (of measured metabolite values); CRLB, Cramer-Rao lower bound for fitting metabolite resonances to their basis set; SNR, signal to noise ratio of singlets in the metabolite spectrum; IP, empirical inflection point separating high- and low-quality measurements (in same units as the quality metric examined). grp = group; Other abbreviations as in Table 1.

a Weighted mean percent difference between patients and controls. See section 2.4 in text.

b Number of outlier datasets for which the 95% CI does not overlap the 95% CI of the pooled data. (Y) or (N) = exclusion of outliers DOES (Y) or DOES NOT (N) change the statistical significance or change a significant effect size by ≥ 1/3.

c Not robust to leave-one-out sensitivity analysis.

In a secondary analysis, a similarly significant effect of measurement quality was seen across the 21 datasets reporting mean CRLB for choline in the MPFC (Table 2). Choline was significantly elevated across 7 datasets reporting mean CRLB ≤ 3% (*g* = +0.29, p =.0012), while no significant effect was seen across 14 datasets reporting mean CRLB > 3% (*g* = +0.03, p =.66) (Table 2). The significant moderating effect of CRLB was robust to leave-out-one analysis and unaffected by exclusion of outliers. Evidence that CRLB quality has a significant influence on MPFC choline effect size independently of COV is presented in Supplemental Results.

Significant quality metric effects on MPFC choline were not observed for 21 datasets reporting FWHM or 23 datasets reporting SNR. However, the qualitative trend for both these metrics was for greater elevations of choline to be seen in higher quality datasets.

For MPFC NAA, a logistic fit to the COV data was significant (r^2^ = 0.47, p <.0001) with an inflection point at COV ≤ 19%. However, the 42 higher quality and the 11 lower quality datasets identified using this threshold did not differ significantly. When six outlier studies were removed, the statistical result became trend-level (p =.068). This trend was not robust to leave-out-one analysis. In a secondary analysis of 27 datasets reporting SNR, a significant logistic fit (r^2^ = 0.76, p <.00001) was observed with an inflection point at mean SNR minus 2 SDs = 12.5 (Table 2). Subgroups defined using this threshold were significantly different (p <.0064). This result was free of small study bias, robust to leave-one-out analysis, and unaffected by exclusion of outliers. NAA was significantly reduced across 15 datasets in which mean SNR minus 2 SDs ≥ 12.5 (*g* = -0.37, p <.00001), while no significant effect was seen across 12 datasets with mean SNR minus 2 SDs < 12.5 (*g* = -0.025, p =.86) (Table 2). Significant quality metric effects were not observed for the 21 datasets reporting FWHM or the 26 datasets reporting mean CRLB. As with SNR, the qualitative trend for COV, CRLB and FWHM was for greater reductions of NAA in higher quality datasets.

For MPFC creatine, no quality metric effects were significant across 38 studies reporting COV, 20 datasets reporting SNR, 17 datasets reporting FWHM, or 16 datasets reporting mean CRLB. For three of the four quality metrics, better measurement quality was associated qualitatively with pooled effect sizes closer to zero for MPFC creatine.

All SNR results reported above were unchanged when restricting the moderator analyses to datasets that used the LCModel method for calculating SNR (k = 23, 19, and 18 for NAA, choline and creatine, respectively).

#### 3.2.2 Measurement quality moderation effects were not significant in other brain regions

In all brain regions other than MPFC, COV was the only measurement quality metric available for ≥ 14 datasets (the minimum needed for the quality metric analysis). Creatine datasets outside of the MPFC all had k < 14 datasets. Thus, measurement quality effects were examined only for COV and only for NAA and/or choline in all other regions. For NAA in the hippocampus, DLPFC, thalamus, basal ganglia and frontal white matter, logistic fits to the COV data were significant and identified COV inflection points at 13.8%, 17.5%, 19.5%, 12.4% and 20.6%, respectively. For all regions except the basal ganglia, studies with lower COV values tended to show greater reductions in NAA. In the basal ganglia, such studies tended to show slight elevations in NAA. However, the moderating effect of COV quality subgroups was significant only for the thalamus, but this effect was not robust to leave-one-out analysis. For choline in the hippocampus, DLPFC, thalamus, and basal ganglia, logistic fits to the COV were not significant, indicating no evidence of data quality thresholds with a significant influence on the pooled effect sizes. For frontal white matter, **t**here were too few choline datasets (k = 12) to perform an analysis of measurement quality effects for choline.

### 3.3 Moderating effects of technical factors

For all regional metabolites with at least 10 datasets available, potential moderating effects of technical factors were examined, including field strength (<3T versus ≥ 3 T) and metabolite normalization method (water versus creatine) using subgroup comparisons, and echo time (as log TE) using *meta*-regression.

#### 3.3.1 Moderating effects of technical factors in hippocampus and frontal white matter

Evidence for moderation by echo time was found for NAA in the hippocampus and frontal white matter. Across all 23 hippocampal NAA studies, *meta*-regression with log TE was not significant (p =.11) but became significant when three outliers were removed (p =.014, Table 3). The reduction in hippocampal NAA was greater in studies using longer echo times. This result, however, was not robust to leave-one-out sensitivity analysis. In secondary analyses, we categorized studies using TE ≤ 35 ms as short TE studies and those using TE > 35 ms as longer TE studies (based on the bimodal distribution of log echo times). Hippocampal NAA was significantly reduced in the 7 longer TE studies (*g* = -0.52, CI = -0.15 to -0.88, p =.0055), but not in the 16 short TE studies (*g* = -0.13, CI = +0.06 to -0.33, p =.19) (Table 3). Since some of the water signal within a voxel may have different T2 relaxation behavior than metabolites, we also conducted secondary analyses separately on water-normalized and creatine-normalized NAA datasets. Across 10 water-normalized datasets, *meta*-regression with log TE was significant (p =.0072), free of small study bias or outliers, and robust to leave-one-out analysis (Table 3). Across 13 creatine-normalized datasets, no effect of log TE on hippocampal NAA effect size was observed (p =.75, Table 3 and Supplemental Figures).

**Table 3:** Significant Technical Moderators.

	Subgroups or Regressor	Datasets	Cases	HC	Effect Size with (95% CI)	P value	Percent Diff a Pt - HC	Heterogeneity I^2^, % P value	number outliers (effect?)^b^
HC NAA	LN TE	23	615	611	NS	0.11	n/a	61.0 < 0.001	3 (Y)
	-Excl outliers	20	520	531	Lower NAA with long TE	**0.013**	n/a	14.8 0.30	NR d
	TE > 35	7	238	232	**-0.52 (-0.88 to -0.15)**	**0.0055**	**−8.9%**	71.7 < 0.001	0
	TE ≤ 35	16	377	379	-0.13 (-0.33 to + 0.06)	0.19	−1.6%	41.1 0.026	0
	LN TE (H_2_O)	10	186	263	Lower NAA with long TE	**0.0072**	n/a	0.0 0.56	0
	LN TE (Cr)	13	429	348	NS	0.75	n/a	76.0 < 0.001	1 (N)
	*All studies*	*23*	*615*	*611*	***-0.26 (-0.46 to -0.07)***	***0.0088***	***−4.4%***	*63.8 < 0.001*	*1 (N)*
									
FrWM NAA	LN TE	14^c^			NS	0.093	n/a	43.1 0.048	1 (Y)
	-Excl outliers	13			Lower NAA with long TE	**0.012**	n/a	10.0 0.30	NR c
	TE > 35	4			**-0.60 (-1.01 to -0.20)**	**0.0036**	**−5.4%**	45.5 0.15	0
	TE ≤ 35	11^c^			**-0.26 (-0.04 to -0.47)**	**0.020**	**−3.0%**	37.9 0.074	0/NR c
	*All studies*	*14*^c^	*455*	*345*	***-0.28 (-0.45 to -0.11)***	***0.0012***	***−3.4%***	*21.8 0.14*	*1 (N)*
									
HC Choline	≥3T v < 3 T	18^c^	538	499	Higher Chol with ≥ 3 T	**0.0037**	n/a	0.0 0.36	0
	Tesla ≥ 3 T	8	254	258	**+0.25 (+0.08 to + 0.43)**	**0.0052**	**+6.1%**	0.0 0.92	0
	Tesla < 3 T	11	299	256	-0.016 (-0.28 to + 0.24)	0.90	−0.1%	53.3 0.021	0
	*All studies*	*19*	553	514	*+0.10 (-0.06 to + 0.27)*	*0.21*	*+2.9%*	*38.7 0.037*	*1 (N)*

Abbreviations: LN TE = natural log of echo time; T = field strength in Tesla; Excl outliers = after excluding datasets identified as outliers in the preceding model; (H_2_O) = water-normed NAA; (Cr) = creatine-normed NAA; Other abbreviations as in Table 1.

a Weighted mean percent difference between patients and controls. See section 2.4 in text.

b Number of outlier datasets for which the 95% CI does not overlap the 95% CI of the pooled data. (Y) or (N) = exclusion of outliers DOES (Y) or DOES NOT (N) change the statistical significance or change a significant effect size by ≥ 1/3.

c One study identified as a source of small study bias was excluded from this model.

d NR = not robust to leave-one-out sensitivity analysis.

Similar evidence for moderation of NAA effect sizes by TE was found for frontal white matter. Across 14 studies free of small study bias, *meta*-regression with log TE was a non-significant trend (p =.09) but became significant when one outlier was removed (p =.012, Table 3). The reduction in frontal white matter NAA was greater in studies using longer echo times. This result, however, was not robust to leave-one-out sensitivity analysis. In secondary analyses, frontal white matter NAA was significantly reduced in the 4 studies using TE > 35 ms (*g* = -0.60, CI = -0.20 to −1.01, p =.0036), as well as in the 11 studies using TE ≤ 35 ms (*g* = -0.26, CI = -0.04 to -0.27, p =.020) (Table 3 and Supplemental Figures). The moderating effect of echo time did not reach significance when either water-normalized (k = 9) or creatine-normalized (k = 6) datasets were analyzed separately.

While elevation of hippocampal choline was not significant in the overall *meta*-analysis, the choline effect size was significantly moderated by field strength. Across 18 studies free of small study bias, the studies conducted at ≥ 3 T showed significantly greater choline elevation than studies conducted at < 3 T (p =.0037) (Table 3). Secondary analysis showed significantly elevated hippocampal choline across the 8 higher field studies (*g* = +0.25, CI = +0.08 to + 0.43, p =.0052) and no significant effect of diagnosis across the 11 lower field studies (*g* = -0.02, CI = -0.28 to + 0.24, p =.90) (Table 3).

No additional significant and robust effects of any technical factors were found for any metabolite in the hippocampus or any other brain regions.

### 3.4 Moderating effects of clinical factors

Potential moderating effects of mean patient age, percent males in the patient group, and percent of patients on antipsychotic medication (using *meta*-regression) and recent onset (<36 months) versus chronic (≥36 months) psychosis (using subgroup comparison) were examined for all regional metabolites with at least 10 datasets available.

#### 3.4.1 Hippocampal NAA is more reduced in studies with more patients on medication

Across 19 studies reporting medication status, *meta*-regression showed that the percent of patients on antipsychotic medication within a study was significantly associated with greater reduction in hippocampal NAA (Table 4). This result was free of small study bias, robust to leave-one-out analysis, and unchanged when outliers were excluded. A secondary analysis showed that across the 11 datasets with ≥ 80% of patients taking antipsychotic medication, the effect size for reduced hippocampal NAA was highly significant (*g* = -0.49, CI = -0.76 to -0.22, p =.0003) (Table 4). Across the six datasets with ≤ 20% of patients taking antipsychotic medication, the effect size for hippocampal NAA was nonsignificant (*g* = +0.09, CI = -0.12 to + 0.30, p =.40) (Table 4).

**Table 4:** Significant Clinical Moderators.

	Subgroups or Regressor	Datasets	Cases	HC	Effect Size with (95% CI)	P value	Percent Diff a Pt - HC	Heterogeneity I^2^, % P value	number outliers (effect?)^b^
HC NAA	% med	19	544	531	Lower NAA with >%med	**0.0032**	n/a	48.0 0.0095	1 (N)
	≥80% med	11	318	300	**-0.49 (-0.76 to -0.22)**	**0.00029**	**−9.5%**	61.3 0.0015	1 (N)
	≤20% med	6	184	203	+0.089 (-0.12 to + 0.30)	0.40	+1.2%	0.0 0.86	0
	*All studies*	*23*	615	611	***-0.26 (-0.46 to -0.07)***	***0.0088***	**−4.4%**	*63.8 < 0.001*	*1 (N)*
MPFC NAA	% med	46	1374	1274	NS	**0.**28	n/a	55.4 < 0.001	7 (Y)
	Excl outliers	39	1286	1268	Lower NAA with >%med	**0.0051**	n/a	0.0 0.85	
	≥80% med	33	988	895	**-0.30 (-0.46 to -0.14)**	**0.0026**	**−3.1%**	42.4 < 0.001	7 (Y)
	-Excl outliers	26	747	728	**-0.44 (-0.55 to -0.34)**	**<0.00001**	**−4.9%**	0.0 0.88	
	≤20% med	8	220	200	-0.15 (-0.34 to + 0.05)	0.14	−0.7%	0.0 0.61	0
	*All studies*	*53*	1527	1435	***-0.225 (-0.34 to -0.11)***	***0.00007***	***−2.2%***	*52.2 0.001*	*7 (Y)*
									
MPFC Choline									
Low COV	% med	34	1086	1011	trend-level	0.079	n/a	33.2 0.02	2 (Y)
	-Excl outliers	32	1038	968	Higher Chol with >%med	**0.020**	n/a	2.9 0.50	
	≥80% med	24	768	699	**+0.29 (+0.14 to + 0.45)**	**0.00023**	**+4.7%**	49.1 0.004	2 (N)
	≤20% med	5	155	135	+0.077 (-0.16 to + 0.31)	0.53	+0.2%	0.0 0.98	0
	*All studies*	*36*	*1121*	*1041*	***+0.22 (+0.11 to + 0.33)***	***0.00015***	***+3.0%***	*36.1 0.012*	*2 (N)*

Abbreviations: % med = percent of patients taking antipsychotic medication; T = field strength in Tesla; Low cov = MPFC choline datasets in the empirically-identified higher measurement quality subgroup as indexed by the coefficient of variation (COV) of choline values (COV ≤ 19%); Other abbreviations as in Table 1.

a Weighted mean percent difference between patients and controls. See section 2.4 in text.

b Number of outlier datasets for which the 95% CI does not overlap the 95% CI of the pooled data. (Y) or (N) = exclusion of outliers DOES (Y) or DOES NOT (N) change the statistical significance or change a significant effect size by ≥ 1/3.

#### 3.4.2 Medication status moderates MPFC NAA and choline effect sizes

Studies of the MPFC demonstrated a similar pattern to the hippocampus, in which studies with more patients on antipsychotic medication had stronger effect sizes for reduced NAA. Although this *meta*-regression was significant only when outlier studies were removed, the result was robust to leave-one-out analyses (Table 4). A follow-up analysis showed that across the 33 datasets with ≥ 80% of patients taking antipsychotic medication, the effect size for reduced MPFC NAA was significant (*g* = -0.30, CI = -0.46 to -0.14, p =.0051). Across the eight datasets with ≤ 20% of patients taking antipsychotic medication, the effect size for MPFC NAA was nonsignificant (*g* = -0.15, CI = -0.34 to + 0.59, p =.14) (Table 4).

No significant effect of any clinical factor was seen for MPFC choline when all studies were included in the analyses. Since measurement quality as indexed by COV was a significant moderator of the elevated choline effect in the MPFC, secondary analyses of potential clinical and technical moderators were conducted on the subset of MPFC choline datasets identified as having higher measurement quality (COV ≤ 19%). Thirty-four of these 36 higher quality datasets reported on patient antipsychotic medication status. Across these studies, the *meta*-regression with percent of medicated patients was significant and robust with two outliers excluded and showed that choline was more strongly elevated in studies with a higher percentage of medicated patients (Table 4). In 24 low COV studies with ≥ 80% medicated patients, MPFC choline was significantly elevated (*g* = +0.29, p =.00023; I^2^ = 49.1). In 5 low COV studies with ≤ 20% medicated patients, no significant difference in MPFC choline was observed (*g* = +0.077, p =.53; I^2^ = 0.0). No other significant and robust effects of clinical or technical moderators were found in this higher measurement quality subset of MPFC choline datasets.

#### 3.4.3 No moderating effects of clinical factors in other brain regions

No other consistent associations with clinical factors were found for NAA, choline or creatine in other brain regions in our primary analyses. We also conducted secondary analyses of NAA restricted to studies including only unmedicated patients. No significant reduction in NAA was seen in any brain region, nor across all regions, in unmedicated patients.

## 4 Discussion

### 4.1 Main findings

In this updated *meta*-analysis of NAA, choline, and creatine levels, we found elevated levels of choline, confirmed previous findings of lower NAA, and found no evidence of abnormal creatine in people with schizophrenia compared to controls. When measured in the MPFC, we found that effect sizes for metabolite abnormalities were significantly moderated by measurement quality such that choline was more elevated and NAA was more reduced in studies with better measurement quality. When measured in the hippocampus and frontal white matter, we observed significant moderation of NAA effect size by echo time. The effect size for hippocampal choline was moderated by field strength. We also found that medication status moderated NAA group differences in the hippocampus, with significantly greater reduction in NAA observed in studies with a higher percentage of patients taking antipsychotic medication. Similar effects of medication status were seen for reduced NAA and elevated choline in the MPFC.

### 4.2 Elevated choline-containing compounds in schizophrenia

For the first time in a comprehensive *meta*-analysis, we demonstrate increased choline in multiple brain areas in patients with schizophrenia compared to healthy volunteers. This effect was significant in MPFC, DLPFC, and BG, and was significant in HC across studies using ≥ 3 Tesla systems. Choline levels measured with MRS are thought to relate to the density of cell membranes, and membrane phospholipid turnover or breakdown (Maddock and Buonocore, 2012). Viewed from this perspective, increased choline could reflect one of several aspects of schizophrenia pathophysiology. Firstly, increased choline may support the reduced neuropil theory of schizophrenia. If the number of neuronal cell bodies in grey matter is the same but the average size of each is reduced, we would expect to see a relative increase in choline-containing compounds due to increased density of neuronal cell bodies and resultant increase in membrane to volume ratios. Secondly, this signal could relate to increased membrane turnover or breakdown of neuronal processes such as dendritic spines. Reduction in dendritic spine density has been postulated to be a key pathophysiological characteristic of schizophrenia (Glantz and Lewis, 2000, Glausier and Lewis, 2013). This process is thought to predominate early in the course of illness. If so, and if the choline increase is related to dendritic spine loss, we would expect it to be more evident in early psychosis. However, we found no evidence that elevated choline was moderated by phase of illness in any brain region. Thirdly, increased choline could indicate phospholipid breakdown due to increased myelin turnover within white matter. No increase in choline, however, was seen in frontal white matter voxels. In contrast, the current analysis suggests that the changes affecting metabolism of mobile choline-containing compounds are primarily located in the basal ganglia, prefrontal grey matter, and the hippocampus**.**

Elevated choline signal may reflect a neuroinflammatory response associated with glial activation. Elevated choline has been observed at the *meta*-analytic level in several disorders associated with neuroinflammation, including HIV infection (Chelala et al., 2020, Dahmani et al., 2021) chronic hepatitis C infection (Oriolo et al., 2018), and type 2 diabetes (Wu et al., 2017). Similar findings have been reported in bipolar (Scotti-Muzzi et al., 2021) and unipolar depression(Riley and Renshaw, 2018, Yildiz-Yesiloglu and Ankerst, 2006). Our finding of significantly increased choline in prefrontal gray matter regions, hippocampus, and basal ganglia is in conceptual agreement with the viewpoint that neuroinflammatory processes may contribute to pathogenesis in schizophrenia (Kirkpatrick and Miller, 2013).

### 4.3 Reduced NAA in schizophrenia

Reduced NAA is the most consistent MRS finding across schizophrenia studies, including in large *meta*-analyses. (Kraguljac et al., 2012, Steen et al., 2005, Whitehurst et al., 2020). Though ubiquitous in neurons and glia, its exact physiological roles are not fully understood. NAA is predominantly synthesized in neuronal mitochondria from acetylation of aspartate by the enzyme *aspartate N-acetyltransferase*. NAA accumulates in neurons to a relatively high concentration, and one of its putative roles is regulation of osmotic balance. Due to its synthesis in neuronal mitochondria, NAA may be regarded as a marker of mitochondrial functional capacity (Maddock and Buonocore, 2012), and thus is regarded by many as a marker of neuronal integrity. Once released into extracellular space, NAA travels down its concentration gradient to oligodendrocytes, where it is broken down by *aspartoacetylase* to release its acetate moiety, which is integral to myelination (Baslow, 2003). An autosomal-recessive disorder in which NAA is not properly catabolized by aspartoacetylase (Canavan disease) results in a fatal disorder marked by CNS dysfunction, deterioration, and abnormal myelination (Baslow and Guilfoyle, 2013). NAA may also correlate with grey matter volume as well as neurocognitive measures such as verbal and spatial reasoning (Paul et al., 2016).

Based on the above, observations of reduced NAA in schizophrenia may reflect decreased neuronal integrity, inefficient mitochondrial function, dysregulated myelin maintenance, and/or altered osmolality. Each of these processes has been implicated in schizophrenia. Reduced integrity or poorer function of glutamatergic neurons could underlie inadequate N-methyl-D-aspartate receptor signaling onto inhibitory neurons, contributing to cognitive and affective symptoms. Mitochondria provide energy for maintenance of spines, establishment of synapses, and vesicular recycling. Interestingly, schizophrenia risk genes identified by large GWAS studies are enriched for mitochondrial genes (Kim et al., 2021, Ripke, S., Neale, B.M., Corvin, A., Walters, J.T.R., Farh, K.-H., Holmans, P.A., Lee, P., Bulik-Sullivan, B., Collier, D.A., Huang, H., Pers, T.H., Agartz, I., Agerbo, E., Albus, M., Alexander, M., Amin, F., Bacanu, S.A., Begemann, M., Jr, R.A.B., Bene, J., Bergen, S.E., Bevilacqua, E., Bigdeli, T.B., Black, D.W., Bruggeman, R., Buccola, N.G., Buckner, R.L., Byerley, W., Cahn, W., Cai, G., Campion, D., Cantor, R.M., Carr, V.J., Carrera, N., Catts, S.V., Chambert, K.D., Chan, R.C.K., Chen, R.Y.L., Chen, E.Y.H., Cheng, W., Cheung, E.F.C., Chong, S.A., Cloninger, C.R., Cohen, D., Cohen, N., Cormican, P., Craddock, N., Crowley, J.J., Curtis, D., Davidson, M., Davis, K.L., Degenhardt, F., Favero, J.D., Demontis, D., Dikeos, D., Dinan, T., Djurovic, S., Donohoe, G., Drapeau, E., Duan, J., Dudbridge, F., Durmishi, N., Eichhammer, P., Eriksson, J., Escott-Price, V., Essioux, L., Fanous, A.H., Farrell, M.S., Frank, J., Franke, L., Freedman, R., Freimer, N.B., Friedl, M., Friedman, J.I., Fromer, M., Genovese, G., Georgieva, L., Giegling, I., Giusti-Rodríguez, P., Godard, S., Goldstein, J.I., Golimbet, V., Gopal, S., Gratten, J., Haan, L. de, Hammer, C., Hamshere, M.L., Hansen, M., Hansen, T., Haroutunian, V., Hartmann, A.M., Henskens, F.A., Herms, S., Hirschhorn, J.N., Hoffmann, P., Hofman, A., Hollegaard, M.V., Hougaard, D.M., Ikeda, M., Joa, I., Julià, A., Kahn, R.S., Kalaydjieva, L., Karachanak-Yankova, S., Karjalainen, J., Kavanagh, D., Keller, M.C., Kennedy, J.L., Khrunin, A., Kim, Y., Klovins, J., Knowles, J.A., Konte, B., Kucinskas, V., Kucinskiene, Z.A., Kuzelova-Ptackova, H., Kähler, A.K., Laurent, C., Keong, J.L.C., Lee, S.H., Legge, S.E., Lerer, B., Li, M., Li, T., Liang, K.-Y., Lieberman, J., Limborska, S., Loughland, C.M., Lubinski, J., Lönnqvist, J., Jr, M.M., Magnusson, P.K.E., Maher, B.S., Maier, W., Mallet, J., Marsal, S., Mattheisen, M., Mattingsdal, M., McCarley, R.W., McDonald, C., McIntosh, A.M., Meier, S., Meijer, C.J., Melegh, B., Melle, I., Mesholam-Gately, R.I., Metspalu, A., Michie, P.T., Milani, L., Milanova, V., Mokrab, Y., Morris, D.W., Mors, O., Murphy, K.C., Murray, R.M., Myin-Germeys, I., Müller-Myhsok, B., Nelis, M., Nenadic, I., Nertney, D.A., Nestadt, G., Nicodemus, K.K., Nikitina-Zake, L., Nisenbaum, L., Nordin, A., O’Callaghan, E., O’Dushlaine, C., O’Neill, F.A., Oh, S.-Y., Olincy, A., Olsen, L., Os, J.V., Pantelis, C., Papadimitriou, G.N., Papiol, S., Parkhomenko, E., Pato, M.T., Paunio, T., Pejovic-Milovancevic, M., Perkins, D.O., Pietiläinen, O., Pimm, J., Pocklington, A.J., Powell, J., Price, A., Pulver, A.E., Purcell, S.M., Quested, D., Rasmussen, H.B., Reichenberg, A., Reimers, M.A., Richards, A.L., Roffman, J.L., Roussos, P., Ruderfer, D.M., Salomaa, V., Sanders, A.R., Schall, U., Schubert, C.R., Schulze, T.G., Schwab, S.G., Scolnick, E.M., Scott, R.J., Seidman, L.J., Shi, J., Sigurdsson, E., Silagadze, T., Silverman, J.M., Sim, K., Slominsky, P., Smoller, J.W., So, H.-C., Spencer, ChrisC.A., Stahl, E.A., Stefansson, H., Steinberg, S., Stogmann, E., Straub, R.E., Strengman, E., Strohmaier, J., Stroup, T.S., Subramaniam, M., Suvisaari, J., Svrakic, D.M., Szatkiewicz, J.P., Söderman, E., Thirumalai, S., Toncheva, D., Tosato, S., Veijola, J., Waddington, J., Walsh, D., Wang, D., Wang, Q., Webb, B.T., Weiser, M., Wildenauer, D.B., Williams, N.M., Williams, S., Witt, S.H., Wolen, A.R., Wong, E.H.M., Wormley, B.K., Xi, H.S., Zai, C.C., Zheng, X., Zimprich, F., Wray, N.R., Stefansson, K., Visscher, P.M., Consortium, W.T.C.-C., Adolfsson, R., Andreassen, O.A., Blackwood, D.H.R., Bramon, E., Buxbaum, J.D., Børglum, A.D., Cichon, S., Darvasi, A., Domenici, E., Ehrenreich, H., Esko, T., Gejman, P.V., Gill, M., Gurling, H., Hultman, C.M., Iwata, N., Jablensky, A.V., Jönsson, E.G., Kendler, K.S., Kirov, G., Knight, J., Lencz, T., Levinson, D.F., Li, Q.S., Liu, J., Malhotra, A.K., McCarroll, S.A., McQuillin, A., Moran, J.L., Mortensen, P.B., Mowry, B.J., Nöthen, M.M., Ophoff, R.A., Owen, M.J., Palotie, A., Pato, C.N., Petryshen, T.L., Posthuma, D., Rietschel, M., Riley, B.P., Rujescu, D., Sham, P.C., Sklar, P., Clair, D.S., Weinberger, D.R., Wendland, J.R., Werge, T., Daly, M.J., Sullivan, P.F., O’Donovan, M.C., 2014). Both *in* vivo and postmortem studies show mitochondrial genes such as cytochrome *c* oxidase and their regulatory genes are down-regulated in patients (Ni and Chung, 2020) and mitochondrial dysfunction has been noted in schizophrenia (Rajasekaran et al., 2015). Finally, mitochondria contribute to oxidative stress through the production of reactive oxygen species.

Our analysis showed that the effect size for reduced NAA in frontal white matter is comparable to the reduction seen in more grey-matter predominant cortical areas, indicating a potential involvement of NAA in white matter dysfunction in schizophrenia. There has been considerable attention paid to oligodendrocyte pathology in schizophrenia (Takahashi et al., 2011). Like neurons, oligodendrocytes are vulnerable to oxidative stress due to the energy demands of homeostatic membrane turnover, which is maintained through adulthood (Valdés-Tovar et al., 2022). In postmortem studies of schizophrenia, oligodendrocyte proteins and their mRNA transcripts are reduced compared to healthy control brain samples, in numerous brain areas (Liu et al., 2022, Valdés-Tovar et al., 2022). These observations may reflect inefficient maintenance of oligodendrocyte homeostasis, a decreased number of oligodendrocytes, or other pathological processes. These changes may underlie established findings of altered connectivity among brain areas in schizophrenia observed with structural imaging and functional brain network analyses (van Dellen et al., 2020, Karlsgodt, 2020).

We observed decreased NAA in all forebrain areas except the basal ganglia, similar to the regional pattern seen qualitatively in the *meta*-analysis by Whitehurst et al. (2020). A secondary analysis showed a significant moderating effect of brain region on NAA effect size when outliers were excluded (Supplemental Table 1a), with the basal ganglia having the smallest effect size. A subsequent comparison of NAA effect sizes in the basal ganglia to each of the other five regions showed that the reduced NAA effect size was greater in MPFC, DLPFC, and thalamus than in the basal ganglia after removal of outliers. These effects were robust to leave-one-out analysis except for the thalamus (Supplemental Table 1b). These findings suggest that the disease-associated factors influencing NAA levels are significantly different in the basal ganglia than in prefrontal cortical regions. Prior studies have shown that increased dopaminergic activity can upregulate aspartate N-acetyltransferase expression in the medium spiny neurons of the nucleus accumbens (Niwa et al., 2007), which in turn can increase NAA synthesis in this ventral striatal structure (Miyamoto 2014). It is possible that increased dopaminergic tone in striatal regions in schizophrenia could account for the selective sparing of the basal ganglia from the otherwise widespread reduction in forebrain NAA observed in this *meta*-analysis. Another possibility is that reduced NAA reflects a disease-related process that is specific to forebrain glutamatergic projection neurons, which are abundant in all regions examined here other than the basal ganglia (where GABAergic projection neurons predominate) (Kreitzer, 2009).

Across brain areas other than the basal ganglia, the weighted mean NAA reductions ranged from 2.0% to 4.4% in individuals with schizophrenia compared to healthy individuals. The reductions were larger when outliers were excluded and reached 9.5% in hippocampus across studies of predominantly medicated patients (Table 1, Table 4). Some individual studies reported group differences of 15%, 18%, and 22% (Bustillo et al., 2010, Natsubori et al., 2014, Yasukawa et al., 2005), while many studies report lower mean differences. Though the mean reductions observed in this *meta*-analysis are modest, it is important to remember that they occur in the context of homeostatic forces which likely produce compensatory effects for disease-related changes. Weighted mean percent reductions in NAA in mild cognitive impairment and multiple sclerosis can provide further context for changes of this scale. A recent *meta*-analysis of metabolite changes in mild cognitive impairment showed decreases of 5.9% and 8.8% in posterior cingulate cortex NAA normalized to creatine and water, respectively (Song et al., 2021). Applying our method of calculating weighted mean percent difference to published *meta*-analytic data (Caramanos et al., 2005) shows that people with multiple sclerosis have a 3.3% mean reduction in NAA in non-lesional white matter, which, despite its name, is known to exhibit axonal loss (Caramanos et al., 2005). These findings suggest that the scale of reduced NAA levels observed in our *meta*-analysis may have clinical and pathophysiological relevance in schizophrenia. Mega-analytic studies of the relationship between clinical symptoms and regional NAA levels in schizophrenia would be informative in this regard. However, the modest size of the average reduction of NAA and the important effects of measurement quality should be taken into account when considering NAA as a potential biomarker or therapeutic target in schizophrenia.

### 4.4 Lack of evidence for abnormal creatine in schizophrenia

Previous studies have suggested the possibility that creatine measured with MRS in brain may be reduced in schizophrenia (Ongür et al., 2009, Tibbo et al., 2013). This issue is important to the field because creatine is frequently used to normalize measurements of other metabolites in the brain. If creatine levels differ between patients and controls, and metabolite levels are normalized to creatine, then one may not know if, for instance, an increase in choline to creatine ratio is due to a true increase in choline, or a decrease in creatine. However, the current *meta*-analysis argues against this concern, as it consistently shows no difference in creatine between patients and healthy volunteers in any of the six brain areas examined. The forest plots for creatine (Supplemental Figures) illustrate that the confidence intervals for the majority of studies crossed the midline, and a similar number of studies demonstrated increased creatine as decreased creatine in schizophrenia. Sensitivity analysis restricted to datasets with high-quality metabolite measurements also failed to find any evidence that brain creatine levels differ between people with schizophrenia and healthy controls.

Another potential concern about creatine normalization is that creatine content may vary more than water content from subject to subject. Thus, creatine normalization might may add more nuisance variance to metabolite estimates than water normalization. If so, the statistical power for observing disease-related abnormalities would be reduced. A counter argument favoring creatine normalization over water normalization is that the latter requires accurate correction for the CSF content of the voxel, a determination which is susceptible to non-trivial measurement error in clinical studies (Dadar, M., Duchesne, S., Group, C.G. and the C.-Q., 2020, Heinen et al., 2016, Wang et al., 2019). Furthermore, subjects in clinical studies may slightly reposition their heads, and thus reposition the voxel, between the time of metabolite measurement and the time of unsuppressed water measurement. These factors would increase nuisance variance in water normalized data but not in creatine normalized data. It is also possible that water content may be abnormal in schizophrenia (Lesh et al., 2019). A normalization method associated with greater nuisance variance would be expected to have higher COV and lower effect size values. The current study found no *meta*-analytic evidence that NAA or choline effect sizes differed significantly between studies normalizing to creatine compared to those normalizing to water. Metabolite COVs were slightly lower overall in studies normalizing to creatine than in studies normalizing to water (Supplemental Table 2). These results support the idea that either creatine or water normalization can reasonably be used in MRS studies of schizophrenia. In some centers, creatine normalization might be preferable, as it precludes the need to precisely correct for CSF within each voxel.

### 4.5 Metabolite measurement quality moderates the MPFC choline and NAA effect sizes

Metabolite measurement quality was shown to have a significant impact on effect sizes for MPFC choline, and in a secondary analysis, on MPFC NAA. A similar relationship between measurement quality and MPFC glutamate effect size in schizophrenia was recently reported by Smucny et al. (2021). NAA and choline are relatively large singlet resonances, while glutamate is a j-coupled multiplet that can be challenging to measure accurately. Although the impact of measurement quality appears to be less pronounced for MPFC choline and NAA than that recently reported for MPFC glutamate, it is nonetheless an important source of heterogeneity across MRS studies of these MPFC metabolites in schizophrenia. It is not certain why measurement quality effects were only evident in the MPFC. However, more than twice as many datasets were included for this region than for any other region. Our procedure for identifying empirical quality thresholds was most sensitive and accurate when based on the large sample of studies available for the MPFC. Statistical power to demonstrate significant quality effects was also greatest for the MPFC.

A surprisingly small percentage of studies reported the quality metrics of CRLB, SNR, and FWHM (approximately one-third, see Supplemental Table 3), despite the ready availability of these metrics from standard modeling software. This prevented us from examining the effect of these direct quality metrics on effect sizes in any brain regions other than the MPFC. COV is an indirect measure of metabolite measurement quality, as it reflects the sum of true variation across subjects plus nuisance variance due to measurement error. While COV can be calculated for any dataset that reports means and standard deviations, it will help advance the field if future studies routinely report all direct measures of both spectrum quality (FWHM and SNR) and precision of metabolite fitting to the basis set (CRLB). Our findings that CRLB and COV moderate MPFC choline effects and SNR moderates NAA effects in the MPFC indicates that optimizing measurement quality will be an important factor in future studies of the pathophysiological significance of these abnormalities.

The current results suggest some metabolite measurement quality thresholds for future studies of schizophrenia. The empirically identified thresholds for MPFC choline were 19% for mean COV and 3% for mean CRLB (Table 2). For MPFC NAA, our analysis identified a quality threshold corresponding to an SNR value of at least 12.5. Studies meeting these quality thresholds showed greater effect sizes and larger weighted mean percent differences than studies not meeting these thresholds (Table 2). Careful attention to factors affecting measurement quality during acquisition (such as shimming and subject motion), unbiased exclusion of distorted spectra and outlier values, and adherence to quality inclusion thresholds that are stricter than those commonly in use may benefit future investigations of the theoretical and clinical significance of metabolite abnormalities in schizophrenia.

### 4.6 Technical MRS moderators

One of the primary aims of this *meta*-analysis was to examine in depth the possibility that T2 relaxation effects may moderate patient versus control subject differences in NAA content. Evidence from prior reports suggests that the T2 relaxation of NAA is faster in patients than control subjects in the hippocampus (Bracken et al., 2013) and frontal white matter (Kuan et al., 2021, Tunc-Skarka et al., 2009). Faster T2 relaxation of NAA in patients means that NAA peaks would decline more quickly in patients, and patient levels of NAA would appear lower when measured with longer echo times. In agreement with these earlier reports, the current study found *meta*-analytic evidence that larger reductions in NAA in patients are reported in studies using longer echo times for both the hippocampus and frontal white matter. These findings suggest that neuropathological abnormalities in people with schizophrenia may include microstructural changes in the hippocampus and frontal white matter that lead to faster T2 relaxation of NAA. A substantial proportion of NAA in frontal white matter and the hippocampus is localized within neuronal axons. One possible mechanism for the moderating effect of echo time in these regions would be reduced axonal volumes, which would increase the frequency of spin–spin interactions between NAA and less mobile macromolecules within axons. This would lead to faster dephasing of the NAA signal and thus faster T2 relaxation. The current finding of larger reductions in NAA signal in patients reported by studies using longer TEs is consistent with reduced axonal volumes in these two regions.

Slower T2 relaxation of the reference compound (water or creatine) used for normalizing NAA values could also account for the moderating effects of TE observed here. In the hippocampus, the effect of TE was most evident in datasets that normalized NAA to water. A similar observation was made in an earlier review by Bracken et al. (2013), who reported that the effect of echo time on reduced NAA in this region was most evident in studies that normalized NAA to water. No similar evidence implicating water-normalized NAA values was evident in the frontal white matter datasets. This may have been due in part to the low number of longer TE studies in that region.

Although this evidence suggests that NAA is subject to abnormally faster T2 relaxation in some regions in schizophrenia, the current findings also show that NAA concentrations is significantly reduced in many regions independent of the effects of echo time. When the *meta*-analysis for each region was repeated including only studies using short TEs (≤35 ms), NAA remained significantly reduced in the MPFC, DLPFC, frontal white matter, and thalamus. Only in the hippocampus did a previously significant NAA reduction fail to reach significance when longer TE studies were excluded (Supplemental Table 4). It is possible that the primary abnormality affecting NAA in the hippocampus is a microstructural change causing faster T2 relaxation. However, relatively few hippocampal studies in this *meta*-analysis demonstrated good quality metrics, and it is possible that as additional good quality studies of this region become available, a significant reduction in hippocampal NAA acquired with short TE will be observed.

Magnetic field strength moderated effect size in the hippocampus for choline, with higher field strength studies associated with stronger group differences in choline. We did not observe field strength effects on any other brain areas or metabolites. The hippocampus is a small brain region with higher vulnerability to susceptibility effects, as a result of its close proximity to sinus air cavities and petrous bone (Bednařík et al., 2015, Choi and Frahm, 1999). For these reasons, investigators often use smaller voxel sizes for studying the hippocampus. It is possible that the higher signal yield from higher field scanners is particularly advantageous for metabolite measurements from the hippocampus.

### 4.7 Clinical moderators

We found that reduced hippocampal NAA in patients was significantly moderated by the percent of patients taking antipsychotic medication. Studies in which a higher percentage of patients were taking antipsychotics reported larger reductions in hippocampal NAA. The same relationship between proportion of medicated patients and reduced NAA was also observed in the MPFC. This latter *meta*-regression, however, was significant and robust only after outlier datasets were excluded. In agreement with Whitehurst et al. (2020), we also found that NAA was not significantly reduced in any brain region, nor across all regions, when the *meta*-analysis was restricted to studies of unmedicated patients.

Several possible factors could contribute to finding a greater reduction of NAA in studies with a higher percentage of medicated patients. A recent *meta*-analysis of MRS studies of NAA conducted before and after treatment (Kubota et al., 2020) and two subsequently published treatment studies (Birur et al., 2020, Li et al., 2022) report no significant effect of short-term or sub-chronic treatment with antipsychotic medication on NAA levels in either the hippocampus or the MPFC. These studies suggest it is unlikely that short term or sub-chronic use of antipsychotic medication has a causal effect of reducing these regional NAA levels in patients with schizophrenia. In the context of these reports, one interpretation of the current results is that antipsychotic medication use is associated with reduced levels of NAA in hippocampus and MPFC, but that the effect is driven primarily by patients who have been chronically taking these medications. Both schizophrenia and antipsychotic medication use are associated with impairments in mitochondrial function. The latter could contribute to the association between medication status and reduced NAA observed here (Casademont et al., 2007, Chan et al., 2020, Turkheimer et al., 2019).

Another possibility is that the medication effects noted here are confounded with illness chronicity effects on NAA levels. Studies of chronic patients typically have a greater proportion of medicated patients than studies of recent onset patients. Although we observed no significant effects of illness chronicity in any brain region or metabolite, a larger sample of datasets including more studies of unmedicated chronic and medicated recent onset patients would be necessary to demonstrate an association with medication status that is statistically independent of illness chronicity.

Since measurement quality moderated choline effect size in the MPFC, we repeated the analysis of clinical (and technical) moderators in the subset of studies identified as having better quality choline measurements based on COV ≤ 19%. This analysis showed that the elevation in MPFC choline was higher in studies with a higher percentage of patients taking antipsychotic medication. The same open questions discussed above regarding medication status and NAA levels, including the role of short term versus long term medication usage and recent onset versus chronic phase of illness apply to the interpretation of this association between medication status and elevated MPFC choline. Further research will be needed to understand the nature and causes of this association.

### 4.8 Strengths and limitations

Strengths of this *meta*-analysis include our application of rigorous quality control procedures including using leave-one-out sensitivity analysis, Egger’s funnel regression test, examination for outliers, and testing for effects of measurement quality on pooled effect sizes. We used a random effects model for the *meta*-analysis, which is more conservative and robust to heterogeneity than a fixed effects model. We set high thresholds for study inclusion, including a minimum of 8 subjects per group (compared to 5 for many *meta*-analyses), and a minimum of 10 studies per brain area for primary *meta*-analytic hypothesis testing and moderator analyses. To reduce variability, we also excluded studies that did not use 1-D, single voxel, MRS pulse sequences, or normalize to either water or creatine. An important strength of this updated *meta*-analysis is the large number of total studies available for screening and inclusion, including numerous new MRS studies. Together, these strengths allowed us to demonstrate for the first time disease-related abnormalities in both NAA and choline across multiple brain regions as well as their moderation by measurement quality, technical factors and medication status.

Limitations of this study include that *meta*-analyses were conducted on only six brain areas (MPFC, DLPFC, HC, BG, thalamus, FrWM). Other regions of interest, such as parietal, occipital, and orbito-frontal cortices and the cerebellum, did not satisfy our requirement for at least 10 datasets per region. The *meta*-analyses presented here are limited to NAA, choline, and creatine. Although other metabolites such as glutamate, GABA, inositol, and glutathione are of interest in schizophrenia, *meta*-analyses of these metabolites have recently been published (Das et al., 2018, Nakahara et al., 2022, Smucny et al., 2021). Although we demonstrated significant effects of data measurement quality in the MPFC, many studies did not report FWHM, SNR, or even CRLB, making it difficult to detect effects of quality metrics in other brain areas. We identified effects of medication status, but we were not able to dissect this further into first- vs. second-generation antipsychotics, take into account the potential effect of mood stabilizers and/or other concomitant medications, or show that the medication status effect was completely independent of chronicity of illness. Without access to patient-level data, we were unable to examine relationships between symptom severity on metabolite levels. Although we found no moderating effect of using water versus creatine for normalization, we were unable to examine the effects of different strategies used for water normalization, including anatomical image acquisition sequence, co-registration and masking method, segmentation algorithm, and assumptions made regarding tissue water content and relaxation characteristics.

### 4.9 Conclusions

These updated *meta*-analyses of the singlet peaks NAA, choline, and creatine provides new insights into brain metabolite abnormalities in schizophrenia with clear demonstration of increased choline and unchanged creatine in patients, along with the well-established reduction in NAA. This work lays a foundation for future investigations into the role of choline-containing metabolites in schizophrenia, possibly related to cell density, membrane turnover, or glial activation. Our findings of unchanged creatine levels and no significant effect of creatine normalization versus water normalization as a moderating factor in the most commonly studied brain regions in schizophrenia support the view that creatine normalization is a reasonable practice in studies of this disorder. We identified a moderating effect of antipsychotic medication on reduced NAA that merits further investigation. We found *meta*-analytic evidence consistent with prior reports of faster T2 relaxation of NAA in hippocampus and frontal white matter, suggesting an altered microenvironment for NAA in schizophrenia. The finding of significant moderating effects of data measurement quality reiterates the importance of reporting quality metrics and may motivate the adoption of measurement quality thresholds in future MRS studies of schizophrenia that are more conservative than those currently in common use.

## Funding sources

This research did not receive any specific funding or grant from agencies in the public, commercial, or not-for-profit sectors.

## Declaration of Competing Interest

The authors declare that they have no known competing financial interests or personal relationships that could have appeared to influence the work reported in this paper.

## Data Availability

Data will be made available on request.
