# Supplementary material for: Meta-analytic evidence of elevated choline, reduced N-acetylaspartate, and normal creatine in schizophrenia and their moderation by measurement quality, echo time, and medication status
Source: Neuroimage Clin. 2023 Jun 27;39:103461. doi: 10.1016/j.nicl.2023.103461 (PMC10509531; doi:10.1016/j.nicl.2023.103461)
Supplement: Supplementary data 1 [file mmc1.pptx]

## Slide 1
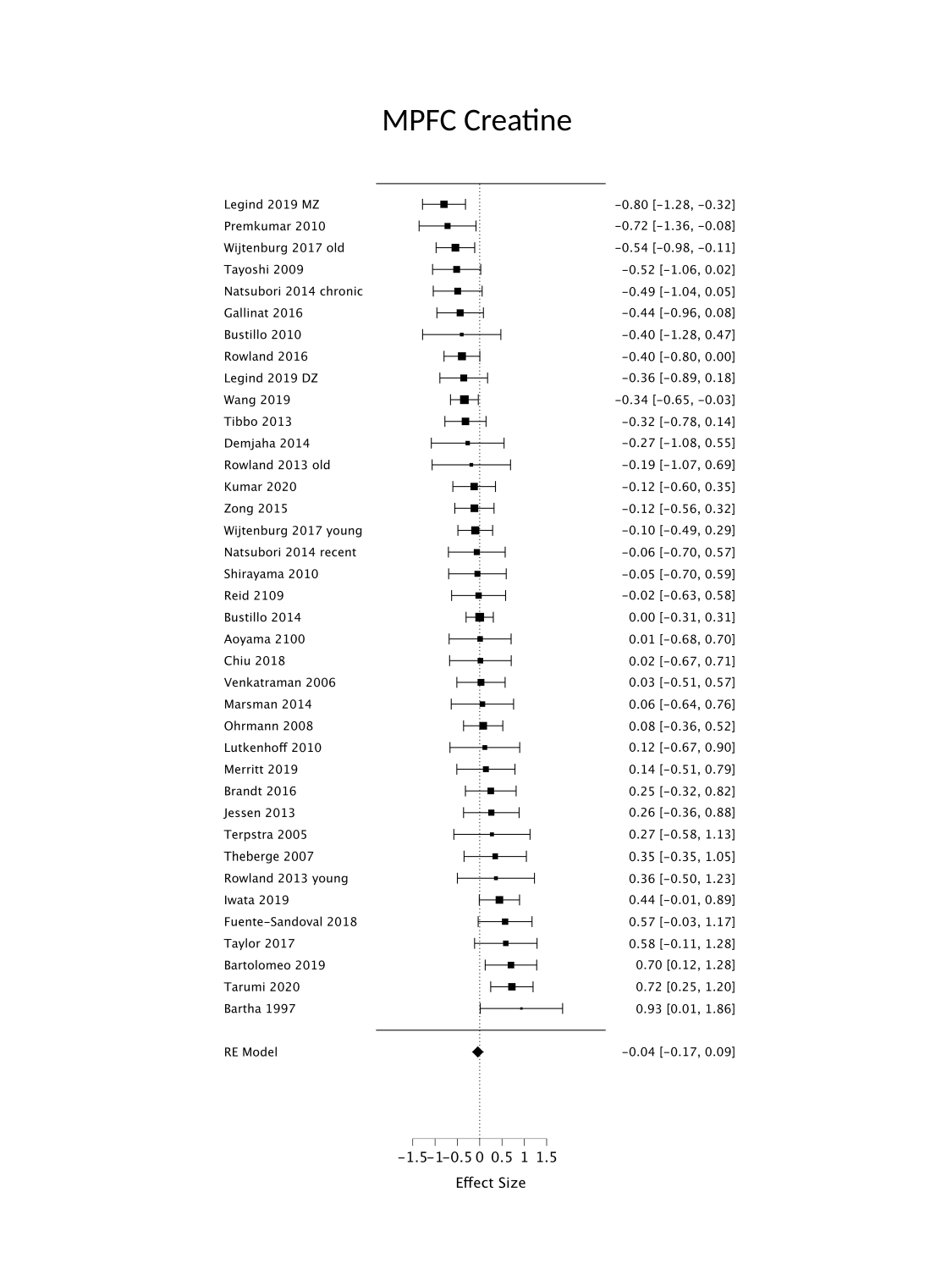

MPFC Creatine

## Slide 2
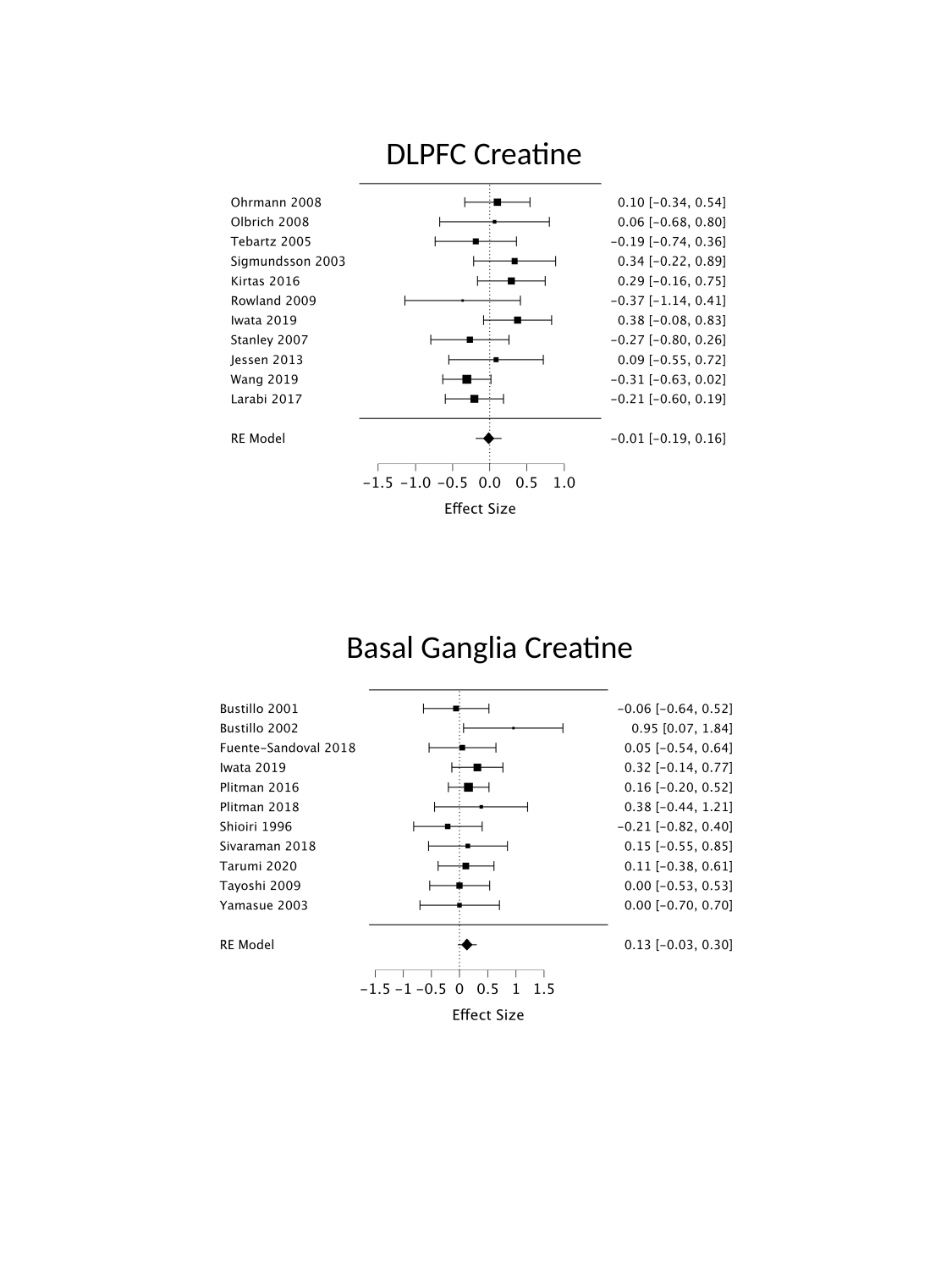

DLPFC Creatine
Basal Ganglia Creatine

## Slide 3
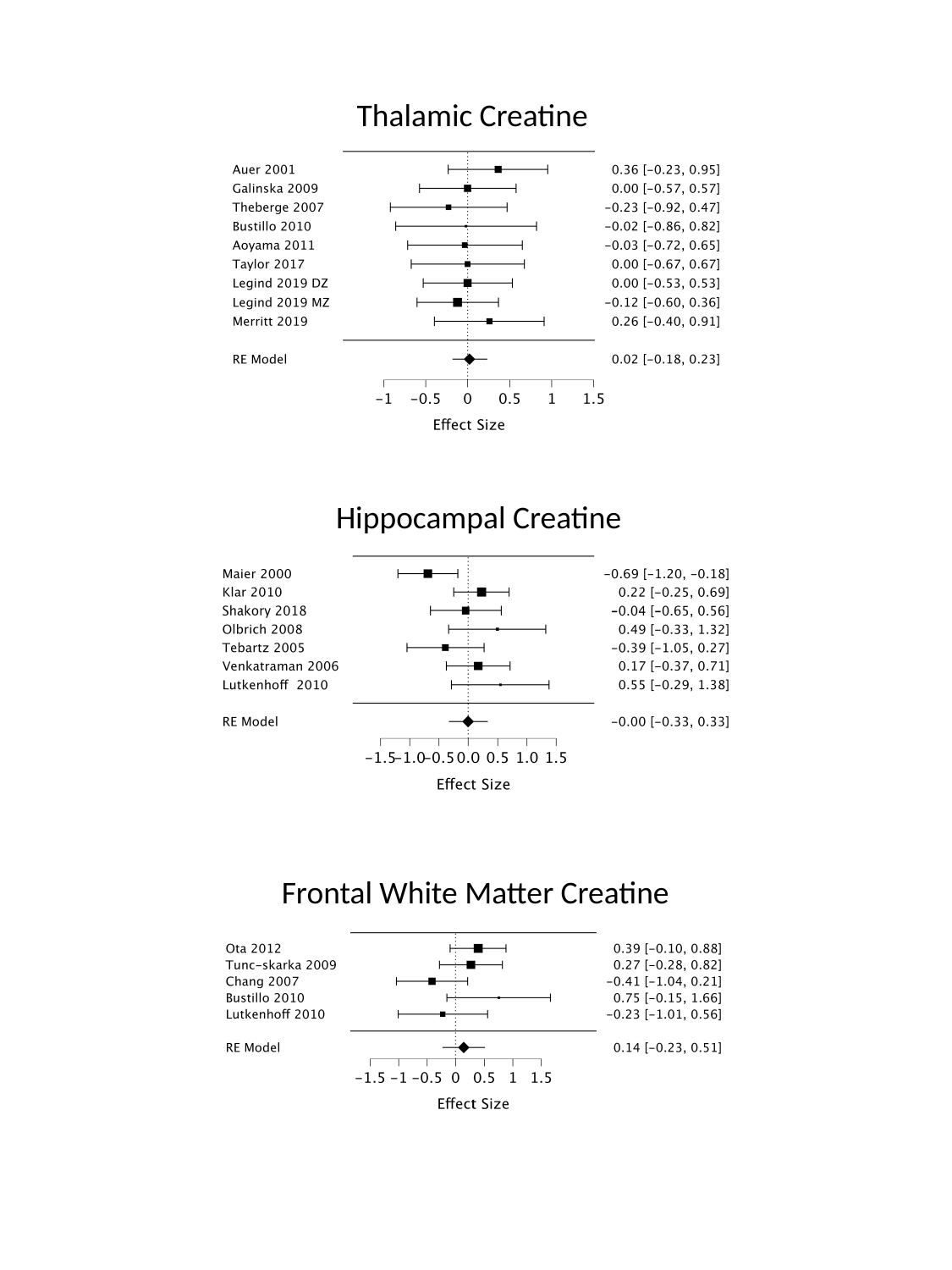

Thalamic Creatine
Hippocampal Creatine
Frontal White Matter Creatine

## Slide 4
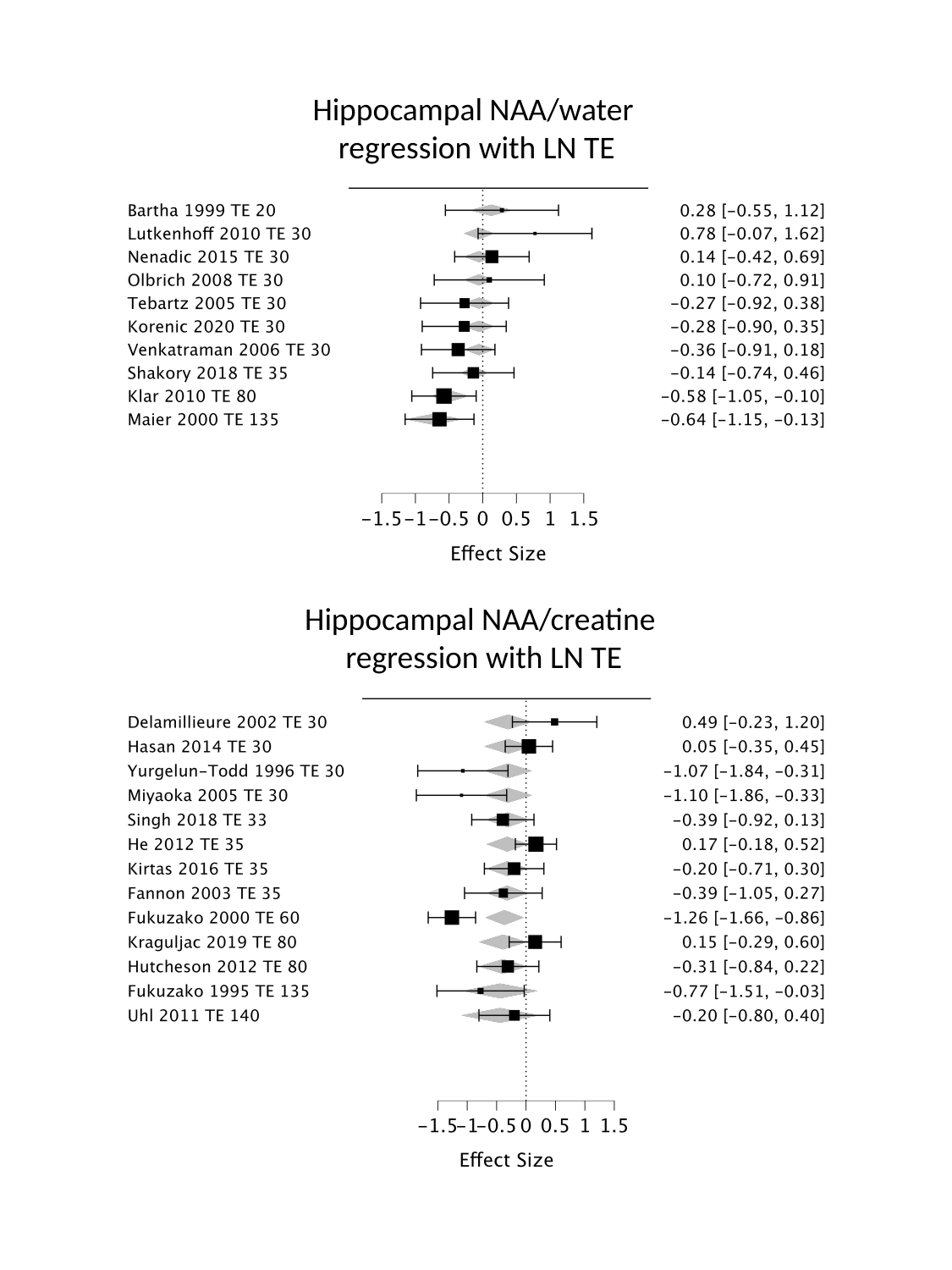

Hippocampal NAA/water
regression with LN TE
Hippocampal NAA/creatine
regression with LN TE

## Slide 5
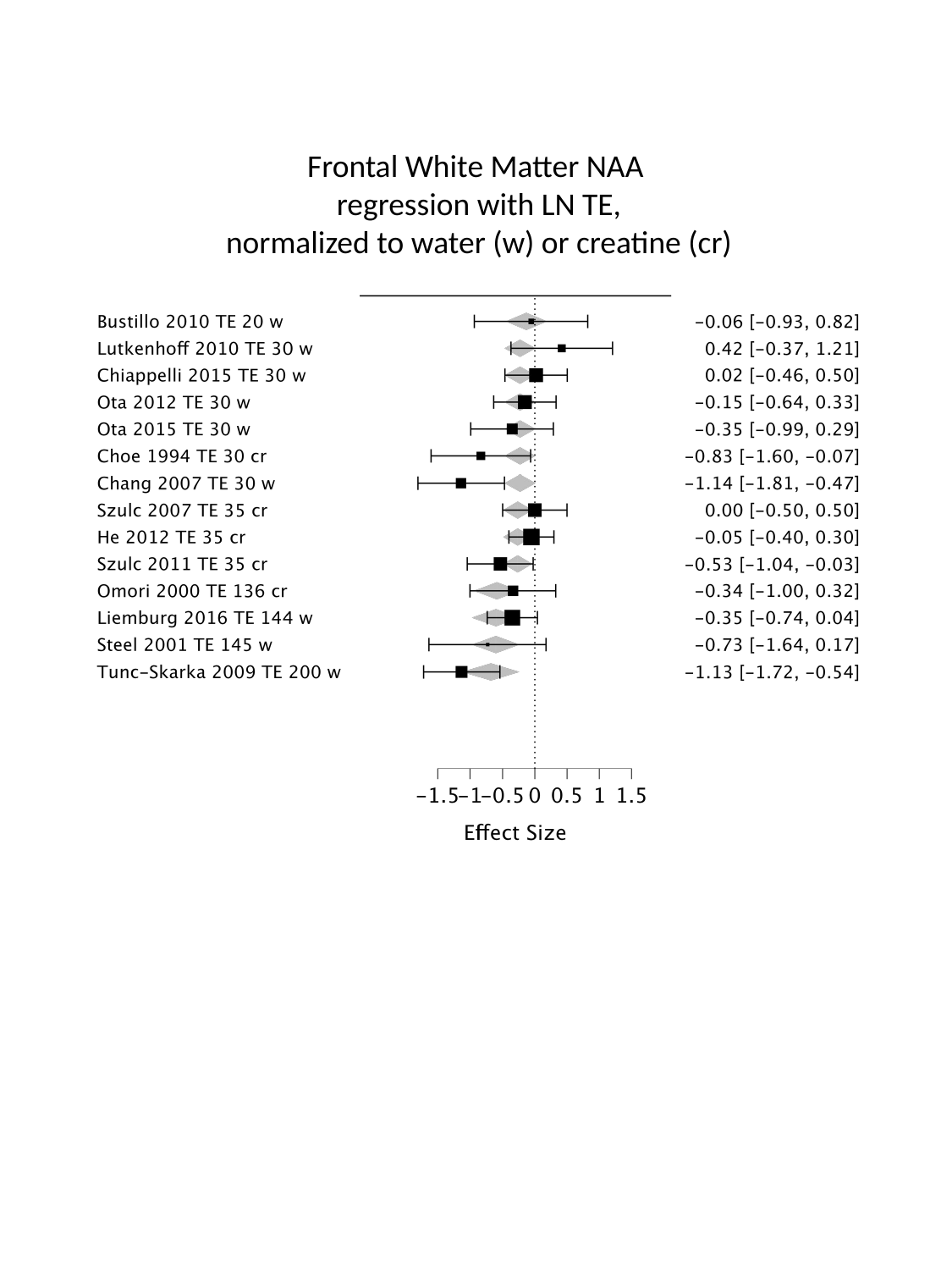

Frontal White Matter NAA
regression with LN TE,
normalized to water (w) or creatine (cr)
