# Supplementary material for: Meta-analytic evidence of elevated choline, reduced N-acetylaspartate, and normal creatine in schizophrenia and their moderation by measurement quality, echo time, and medication status
Source: Neuroimage Clin. 2023 Jun 27;39:103461. doi: 10.1016/j.nicl.2023.103461 (PMC10509531; doi:10.1016/j.nicl.2023.103461)
Supplement: Supplementary data 2 [file mmc2.docx]

Supplemental Materials for:

"Meta-analytic evidence of elevated choline, reduced N-acetylaspartate, and normal creatine in schizophrenia and their moderation by measurement quality, echo time, and medication status"

Yvonne S. Yang, MD, PhD, Jason Smucny, PhD, Huailin Zhang, MD, Richard J. Maddock, MD

Document 1: Supplemental Methods, Results and Tables

Page 1: Table of Contents

Page 2: Supplemental Methods

Page 3: Supplemental Results

Page 4: Supplemental Tables 1a and 1b - NAA in basal ganglia vs. other regions

Page 5: Supplemental Table 2 - Mean COVs for water and creatine normalized values in each region

Page 6: Supplemental Table 3 - Percentage of studies reporting quality metrics by region

Page 7: Supplemental Table 4 - NAA meta-analytic results restricted to TE ≤ 35 for all regions

Document 2: Supplemental Figures

Pages 1-3: Forest plots of Creatine effects in six brain regions

Pages 4-5: Forest plots for TE moderation of NAA effects in hippocampus and frontal white matter

**Supplemental Methods**

Empirical method for identifying quality thresholds.

We examined four metrics sensitive to the quality of the NAA, choline and creatine measurements for each study: mean COV for metabolite values, mean Cramer-Rao lower bound (CRLB) for metabolite fits, mean + 2 SD for singlet line width (FWHM), and mean - 2 SD for singlet signal-to-noise ratio (SNR). For each metric, we averaged the values for the patient and control groups. We hypothesized that metabolite measurement quality would have a moderating effect on the meta-analytic results across studies comparing schizophrenia patients to healthy volunteers. Formally, we hypothesized there was a quality threshold T, for which the meta-analytic result would be significantly stronger in studies surpassing T than for those falling short of T. To identify the quality threshold T in an unbiased manner, we first ranked the studies for each metric. We then calculated the inverse variance-weighted pooled effect sizes (as Hedge’s *g*) from a moving subsample of k’ studies, where k’ = total k/5 or k’ = 7, whichever is greater. The moving sub-meta-analyses ran from the k’ lowest quality studies to the k’ highest quality studies for each quality metric (analogous to a moving average). A best-fitting, 4-parameter, logistic function was fit to this series of pooled effect sizes using the computational resource at <https://findcurves.com/> using the following equation:

Y = d + ((a – d)/(1+(X/c)^b))

Where Y = the pooled effect size (of k’ studies) and X = the rank of the set of k’ adjacent studies for the quality metric being examined. The best fitting four parameters (a, b, c, and d) for each quality metric is shown below for regional metabolites demonstrating significant moderating effects of measurement quality for that quality metric.

|  | a | b | c | d | IP (*g*) | T (metric) |
| --- | --- | --- | --- | --- | --- | --- |
| **MPFC Choline** |  |  |  |  |  |  |
| COV | -.116 | 14.79 | 12.27 | +.248 | +.066 | 19.0% |
| CRLB | +.122 | 25.71 | 11.80 | +.278 | +.200 | 3.0% |
|  |  |  |  |  |  |  |
| **MPFC NAA** |  |  |  |  |  |  |
| SNR | -0.079 | 26.14 | 12.41 | -0.398 | -.239 | 12.5 |
|  |  |  |  |  |  |  |

Parameter “a” is the asymptote of the pooled effect size for the lowest quality datasets, and parameter “d” is the asymptote of the pooled effect size for the highest quality datasets for each metric. These best fitting parameters were used to generate a logistic transform of the ranks of each quality metric. The empirical quality threshold T was identified by the inflection point (IP) in the logistic transform curve. The inflection point is the value of *g* at the midpoint between parameters “a” and “d” (thus IP = (a + d)/2). This point IP was used to stratify studies into low and high quality subgroups for each metric. All studies included in a set of k’ ranked studies for which the moving pooled effect size was to the right of IP (more negative for NAA and more positive for choline) were stratified into the high quality subgroup for that quality metric. The least good quality metric value among studies in the high quality subgroup was defined as the empirical quality threshold (T). All studies with a quality metric value worse than T were stratified into the lower quality subgroup. The units of T are the same as the units of the quality metric.

**Supplemental Results**

Evidence that CRLB influences MPFC choline effect size independently of COV

COV and CRLB may index different aspects of choline measurement quality, since combining the two metrics had additive effects. In a subgroup comparison restricted to the 36 datasets with COV ≤ 19%, 12 studies with CRLB > 3% had a smaller pooled effect size compared to the remaining 24 datasets with COV ≤ 19% (Q= 5.07, p = .024; I^2^ = 20.4, p = .049). This effect became more significant and robust to leave-one-out analysis when two outliers (both with 99% CI non-overlap) were excluded (Q = 14.0, p = .00019; I^2^ = 0.0, p = .91). Across the 12 studies with higher COV quality but lower CRLB quality, MPFC choline was not significantly different between groups (g = +.01, p = .90; I^2^ = 0.0, p = .99). Across the remaining 24 higher quality COV studies, MPFC choline was significantly elevated in patients (g = +.30, p = .00001; I^2^ = 27.6, p = .13). This result was unchanged by exclusion of the single outlier and robust to leave-one-out analysis.

Supplemental Table 1a. Main effect of brain region on patient vs. control NAA effect size

|  | Factor | Datasets | Pts | HC | Direction  of Effect | P value | Heterogeneity  I^2^, % P value | number  outliers  (effect?) ^a^ |
| --- | --- | --- | --- | --- | --- | --- | --- | --- |
| NAA | Brain regions (6) | 154 | 4445 | 3934 |  | .45 | 51.2 <.0001 | 15 (Y) |
|  | -Excl. outliers | 139 | 3991 | 3607 | **BG < others** | **.019** | 37.0 .068 |  |

Supplemental Table 1b. Comparison of NAA effect in basal ganglia (BG) to each other brain region

| Reference  Region | Region  Compared | Datasets | BG Effect Size  (95% CI) | Comp Effect Size (95% CI) | P value | Heterogeneity  I^2^, % P value | number  outliers  (effect?)^a^ |
| --- | --- | --- | --- | --- | --- | --- | --- |
| BG  NAA | vs. MPFC | 73 | -.07 (-.20 to +.06) | -.22 (-.34 to -.11) | .14 | 40.7 .0003 | 7 (Y) |
|  | -Excl outlier | 66 | -.07 (-.20 to +.06) | **-.32 (-.39 to -.24)** | **.0013** | 0.0 .71 |  |
|  | vs. DLPFC | 42 | -.07 (-.20 to +.06) | -.27 (-.46 to -.08) | .10 | 42.4 .0003 | 2 (Y) |
|  | -Excl outlier | 40 | -.07 (-.20 to +.06) | **-.36 (-.53 to -.19)** | **.0034** | 18.2 .15 |  |
|  | vs. Thal | 41 | -.07 (-.20 to +.06) | -.23 (-.40 to -.07) | .069 | 11.4 .26 | 1 (Y) |
|  | -Excl outlier | 40 | -.07 (-.20 to +.06) | **-.28 (-.43 to -.12)** | **.018** | 2.5 .50 | NR |
|  | vs. FrWM | 34 | -.07 (-.20 to +.06) | **-.30 (-.48 to -.12)** | **.031** | 0.0 .47 | 1 (Y) |
|  | -Excl outlier | 33 | -.07 (-.20 to +.06) | -.24 (-.39 to -.09) | .089 | 0.0 .75 |  |
|  | vs. HC | 43 | -.07 (-.20 to +.06) | -.26 (-.46 to -.07) | **.**13 | 44.9 .0006 | 2 (N) |
|  |  |  |  |  |  |  |  |
|  |  |  |  |  |  |  |  |

**^a^** Number of outlier datasets for which the 95% CI does not overlap the 95% CI of the pooled data. (Y) or (N) = exclusion of outliers DOES (Y) or DOES NOT (N) change the statistical significance or change a significant effect size by ≥ 1/3.

Abbreviations: Comp Effect Size = effect size for comparison region; P value = significance of contrast between BG and comparison region; NR = not robust to leave-one-out analysis; Excl outlier = after excluding datasets identified as outliers in the preceding model; other abbreviations as in Table 1.

Supplemental Table 2. Water vs. creatine normalization: Effects on NAA and Choline COV values

|  | Region | K  water | COV  water |  | K  creatine | COV  creatine |  | difference  w - c |
| --- | --- | --- | --- | --- | --- | --- | --- | --- |
| NAA | MPFC | 37 | 0.132 |  | 16 | .109 |  | .024 |
|  | HC | 10 | .231 |  | 13 | .145 |  | .085 |
|  | DLPFC | 12 | .148 |  | 10 | .187 |  | -.040 |
|  | Thal | 11 | .106 |  | 10 | .134 |  | -.029 |
|  | BG | 12 | .111 |  | 9 | .193 |  | -.083 |
|  | FrWM | 9 | .129 |  | 6 | .157 |  | -.029 |
|  | wt. mean ^a^ | 91 | .139 |  | 64 | .149 |  | -.003 |
|  |  |  |  |  |  |  |  |  |
| Choline | MPFC | 35 | .185 |  | 13 | .175 |  | .011 |
|  | HC | 8 | .391 |  | 13 | .169 |  | .221 |
|  | DLPFC | 10 | .235 |  | 7 | .225 |  | .010 |
|  | Thal | 8 | .154 |  | 10 | .152 |  | .001 |
|  | BG | 11 | .154 |  | 8 | .137 |  | .017 |
|  | FrWM | 7 | .186 |  | 5 | .145 |  | .041 |
|  | wt. mean ^a^ | 79 | .205 |  | 56 | .168 |  | .046 |
| Overall | wt. mean ^b^ | 170 | .169 |  | 120 | .158 |  | .020 |

^a^ mean across all regions for one metabolite, weighted by the number of datasets

^b^ mean across all regions and both metabolites, weighted by the number of datasets

COV = coefficient of variation (SD/mean) averaged across patient and control samples within a dataset, then averaged across datasets within a region; K = number of datasets; w = water-normalized; c = creatine-normalized; other abbreviations as in Table 1.

Supplemental Table 3. Frequency of reporting of quality metrics

|  | Region | K | CRLB  # (%) | SNR  # (%) | FWHM  # (%) |
| --- | --- | --- | --- | --- | --- |
| NAA | MPFC | 53 | 26 (49) | 27 (51) | 20 (38) |
|  | HC | 23 | 5 (22) | 5 (22) | 3 (13) |
|  | DLPFC | 22 | 3 (14) | 4 (18) | 3 (14) |
|  | Thal | 21 | 6 (29) | 6 (29) | 5 (24) |
|  | BG | 20 | 4 (20) | 8 (40) | 4 (20) |
|  | FrWM | 15 | 4 (27) | 3 (20) | 3 (20) |
|  | all regions | 154 | 48 (31) | 53 (34) | 38 (25) |
|  |  |  |  |  |  |
| Choline | MPFC | 48 | 21 (44) | 23 (48) | 18 (38) |
|  | HC | 19 | 5 (26) | 5 (26) | 3 (16) |
|  | DLPFC | 16 | 3 (19) | 4 (25) | 3 (19) |
|  | Thal | 18 | 6 (33) | 4 (22) | 3 (17) |
|  | BG | 18 | 4 (22) | 7 (39) | 3 (17) |
|  | FrWM | 12 | 4 (33) | 2 (17) | 2 (17) |
|  | all regions | 131 | 43 (33) | 45 (34) | 32 (24) |
| Creatine | MPFC | 38 | 16 (42) | 20 (53) | 16 (42) |
|  | HC | 7 | 3 (43) | 2 (29) | 2 (29) |
|  | DLPFC | 11 | 3 (27) | 4 (36) | 3 (27) |
|  | Thal | 9 | 5 (56) | 4 (44) | 3 (33) |
|  | BG | 11 | 3 (27) | 6 (55) | 3 (27) |
|  | FrWM | 5 | 2 (40) | 1 (20) | 1 (20) |
|  | all regions | 81 | 32 (40) | 37 (47) | 28 (35) |
| Overall | all regions & metabolites | 366 | 123 (34) | 135 (37) | 98 (27) |

K = number of datasets; # = number of datasets reporting the metric; CRLB = Cramer-Rao lower bound; SNR = signal to noise ratio; FWHM = full width at half maximum of NAA singlet peak; other abbreviations as in Table 1.

Supplemental Table 4. Meta-analytic Results for NAA from Datasets Acquired with TE ≤ 35 msec

|  | Region | K | Pts | | HC | Effect Size  (95% CI) | P value | Heterogeneity  I^2^, % P value | number  outliers  (effect?) ^a^ |
| --- | --- | --- | --- | --- | --- | --- | --- | --- | --- |
| NAA | MPFC | 40 | | 1103 | 1041 | **-.22 (-.36 to -.08)** | **.0023** | 58.1 <.001 | 4 (N) |
|  | -Excl. outliers | 36 | | 929 | 948 | **-.28 (-.39 to -.17)** | **<.00001** | 24.6 .09 |  |
|  | HC | 16 | | 377 | 379 | -.13 (-.33 to +.06) | .19 | 41.1 .026 | 1 (N) |
|  | DLPFC | 12 | | 439 | 409 | -.24 (-.51 to +.04) | .096 | 71.5 <.001 | 1 (Y) |
|  | -Excl. outliers | 11 | | 375 | 383 | **-.32 (-.58 to -.06)** | **.014** | 62.4 .014 |  |
|  | Thal | 17 | | 474 | 455 | **-.26 (-.42 to -.11)** | **.0011** | 23.2 .23 | 0 |
|  | BG | 11 | | 363 | 264 | .00 (-.16 to +.16) | .99 | 0.0 .83 | 0 |
|  | FrWM | 10 ^b^ | 336 | | 283 | **-.28 (-.51 to -.05)** | **.016** | 45.2 .046 | 0 |

^a^ Number of outlier datasets for which the 95% CI does not overlap the 95% CI of the pooled data. (Y) or (N) = exclusion of outliers DOES (Y) or DOES NOT (N) change the statistical significance or change a significant effect size by ≥ 1/3.

^b^ One study identified as a source of small study bias was excluded from this model.

Abbreviations: K = number of datasets; Pts = patients; HC = healthy controls; Excl. = after excluding; other abbreviations as in Table 1.
